# Socioeconomic Indicators of Treatment Prognosis for Adults With Depression: A Systematic Review and Individual Patient Data Meta-analysis

**DOI:** 10.1001/jamapsychiatry.2022.0100

**Published:** 2022-03-09

**Authors:** Joshua E. J. Buckman, Rob Saunders, Joshua Stott, Zachary D. Cohen, Laura-Louise Arundell, Thalia C. Eley, Steven D. Hollon, Tony Kendrick, Gareth Ambler, Edward Watkins, Simon Gilbody, David Kessler, Nicola Wiles, David Richards, Sally Brabyn, Elizabeth Littlewood, Robert J. DeRubeis, Glyn Lewis, Stephen Pilling

**Affiliations:** 1 Centre for Outcomes Research and Effectiveness (CORE), Research Department of Clinical, Educational & Health Psychology, University College London, London, United Kingdom; 2 iCope Camden & Islington NHS Foundation Trust, St Pancras Hospital, London, United Kingdom; 3 Department of Psychiatry, University of California, Los Angeles; 4 Social, Genetic and Developmental Psychiatry Centre, Institute of Psychiatry, Psychology & Neuroscience, King’s College London, London, United Kingdom; 5 Department of Psychology, Vanderbilt University, Nashville, Tennessee; 6 Primary Care, Population Sciences and Medical Education, Faculty of Medicine, University of Southampton, Southampton, United Kingdom; 7 Statistical Science, University College London, London, United Kingdom; 8 Department of Psychology, University of Exeter, Exeter, United Kingdom; 9 Department of Health Sciences, University of York, York, United Kingdom; 10 Centre for Academic Mental Health, Population Health Sciences, Bristol Medical School, University of Bristol, Bristol, United Kingdom; 11 Centre for Academic Primary Care, Population Health Sciences, Bristol Medical School, University of Bristol, Bristol, United Kingdom; 12 Institute of Health Research, University of Exeter College of Medicine and Health, Exeter, United Kingdom; 13 Department of Health and Caring Sciences, Western Norway University of Applied Sciences, Bergen, Norway; 14 University of Pennsylvania College of Arts and Sciences, Department of Psychology, Philadelphia; 15 Division of Psychiatry, University College London, London, United Kingdom; 16 Camden & Islington NHS Foundation Trust, London, United Kingdom

## Abstract

**Importance:**

Socioeconomic factors are associated with the prevalence of depression, but their associations with prognosis are unknown. Understanding this association would aid in the clinical management of depression.

**Objective:**

To determine whether employment status, financial strain, housing status, and educational attainment inform prognosis for adults treated for depression in primary care, independent of treatment and after accounting for clinical prognostic factors.

**Data Sources:**

The Embase, International Pharmaceutical Abstracts, MEDLINE, PsycINFO, and Cochrane (CENTRAL) databases were searched from database inception to October 8, 2021.

**Study Selection:**

Inclusion criteria were as follows: randomized clinical trials that used the Revised Clinical Interview Schedule (CIS-R; the most common comprehensive screening and diagnostic measure of depressive and anxiety symptoms in primary care randomized clinical trials), measured socioeconomic factors at baseline, and sampled patients with unipolar depression who sought treatment for depression from general physicians/practitioners or who scored 12 or more points on the CIS-R. Exclusion criteria included patients with depression secondary to a personality or psychotic disorder or neurologic condition, studies of bipolar or psychotic depression, studies that included children or adolescents, and feasibility studies. Studies were independently assessed against inclusion and exclusion criteria by 2 reviewers.

**Data Extraction and Synthesis:**

Data were extracted and cleaned by data managers for each included study, further cleaned by multiple reviewers, and cross-checked by study chief investigators. Risk of bias and quality were assessed using the Quality in Prognosis Studies (QUIPS) and Grading of Recommendations, Assessment, Development and Evaluation (GRADE) tools, respectively. This study followed the Preferred Reporting Items for Systematic Reviews and Meta-analyses–Individual Participant Data (PRISMA-IPD) reporting guidelines.

**Main Outcomes and Measures:**

Depressive symptoms at 3 to 4 months after baseline.

**Results:**

This systematic review and individual patient data meta-analysis identified 9 eligible studies that provided individual patient data for 4864 patients (mean [SD] age, 42.5 (14.0) years; 3279 women [67.4%]). The 2-stage random-effects meta-analysis end point depressive symptom scale scores were 28% (95% CI, 20%-36%) higher for unemployed patients than for employed patients and 18% (95% CI, 6%-30%) lower for patients who were homeowners than for patients living with family or friends, in hostels, or homeless, which were equivalent to 4.2 points (95% CI, 3.6-6.2 points) and 2.9 points (95% CI, 1.1-4.9 points) on the Beck Depression Inventory II, respectively. Financial strain and educational attainment were associated with prognosis independent of treatment, but unlike employment and housing status, there was little evidence of associations after adjusting for clinical prognostic factors.

**Conclusions and Relevance:**

Results of this systematic review and meta-analysis revealed that unemployment was associated with a poor prognosis whereas home ownership was associated with improved prognosis. These differences were clinically important and independent of the type of treatment received. Interventions that address employment or housing difficulties could improve outcomes for patients with depression.

## Key Points

**Question:**

Are socioeconomic factors associated with depression treatment outcomes regardless of treatment type?

**Findings:**

In this systematic review and meta-analysis that included 9 studies with 4864 participants, socioeconomic disadvantage in employment and housing were associated with worse prognosis outcomes regardless of treatment type and after adjusting for clinical prognostic factors.

**Meaning:**

Accessible information on employment and housing status can inform the intensity of treatment to manage depression and referrals for specialist support; addressing employment and housing needs may make it easier for patients to engage in and achieve better outcomes from treatment for depression.

## Teaser

This systematic review and meta-analysis investigates the socioeconomic factors associated with depression treatment outcome regardless of treatment type.

## Introduction

Many treatments for depression are effective, yet half of all patients do not recover with the first treatment they receive.^1^ This can lead to disengagement and poor long-term outcomes.^2^ Recently, studies have begun to report on pretreatment characteristics associated with prognosis regardless of treatment type, providing patients and clinicians with desired information^3^ and informing assessments and clinical decision-making before a choice of treatment is made.^4^ These include the severity of depressive symptoms; the duration of depression, comorbid anxiety, and comorbid panic disorder; a history of antidepressant treatment^4,5^; marital status^6^; and social support.^7^ Despite such knowledge, most of the variance in prognosis for patients with depression remains unexplained, reflecting the field’s limited knowledge of how patients respond to treatment.^5^

Socioeconomic factors have been associated with increased prevalence of depression^8,9^; however, associations with prognosis have rarely been investigated. In a meta-review, only 2 systematic reviews reported on these associations.^4^ One review^10^ found 2 studies (284 patients combined) that reported that a patient history of more years of education was associated with a favorable prognosis. Further, the review found 1 study (92 patients) that reported homeownership was associated with a more favorable depression prognosis. Another review^11^ found contradictory outcomes across 2 high-quality and 5 lower-quality primary studies, making it difficult to draw conclusions on the association between socioeconomic factors and prognosis for those with depression. Other studies have shown that employment status and educational attainment are associated with outcomes but only investigated this for people treated with citalopram.^12,13^ Collectively, these studies have only addressed a limited range of socioeconomic factors, and crucially, none have adjusted for the outcomes of known clinical prognostic factors. Therefore, the clinical value of using socioeconomic factors to improve prognostication beyond these is unknown.^4,14^ Further, each of the studies either focused on a single type of treatment (eg, specific antidepressants) or studied community samples where treatment was not sought, or details on treatments were poorly described or unknown.^4^ This limits generalizability, particularly to primary care, which is both a common route into treatment and psychiatric care, and where there are typically multiple treatment options.^15,16^ This study, therefore, aimed to investigate: (1) the associations between a range of socioeconomic factors (eg, employment status, financial strain, housing status, and level of educational attainment) and prognosis for adults with depression in primary care, independent of treatment and (2) whether these factors add to knowledge of prognosis after accounting for other known prognostic factors.

## Methods

### Identification and Selection of Studies

This systematic review with individual patient data meta-analysis was reported in accordance with the Preferred Reporting Items for Systematic Reviews and Meta-analyses–Individual Participant Data (PRISMA-IPD)^17^ reporting guidelines (eAppendix in the Supplement). Searches were reported in accordance with the PRISMA-S extension for systematic reviews.^18^ The search strategy and preregistered methods can be found on PROSPERO^19^ and in a general protocol^20^ that was reported in accordance with the PRISMA-P extension for the reporting of systematic review protocols.^21^ eTables 1, 2, 6, and 7 in the Supplement contain details of protocol development, scoping searches, and rationale. Additional methods for the specific data analysis for this study were also preregistered.^22^All included studies were granted ethical approvals by the NHS Research Ethics Committees (eTable 3 in the Supplement). Written informed consent was obtained from all patients. No additional NHS ethical approval was required for this study.

Full searches were conducted on Embase, International Pharmaceutical Abstracts, MEDLINE, PsycINFO, and Cochrane (CENTRAL) from database inception to October 8, 2021 (eTable 1 in the Supplement). Reference lists of returned studies were hand searched, and experts were contacted for unpublished or missed studies. A single reviewer (J.E.J.B.) screened titles and abstracts for eligible studies; these were read in full and judged against inclusion and exclusion criteria by 2 reviewers (J.E.J.B., G.L.) with consensus meetings with a third reviewer (S.P.) to resolve discrepancies.

### Inclusion and Exclusion Criteria

Individual patient data were sought for participants in studies that were randomized clinical trials (RCTs) of participants aged 16 years and older with unipolar depression, had at least 1 active treatment group, had assessed at least 1 socioeconomic factor, and had used the Revised Clinical Interview Schedule (CIS-R) at baseline to measure depressive and anxiety symptoms and chronicity and provide diagnoses. Also included were individual patient data from studies of patients who sought treatment for depression, had a CIS-R score of 12 points or greater, or were recruited from primary care. This ensured that all studies had data available on the key depression disorder characteristics such that any associations found here could inform prognosis over and above those factors that are or should be routinely assessed in clinic pretreatment.^4^ Studies were excluded if they included patients with depression secondary to a personality or psychotic disorder or neurologic condition; if they evaluated adults with bipolar or psychotic depression; and if they included children or adolescents, were feasibility studies, or investigated just 1 socioeconomic group.

### Measures

The CIS-R^23^ was used at baseline in all studies; the CIS-R screens for symptoms and duration of depression and a range of anxiety symptoms, and it provides diagnoses using *International Statistical Classification of Diseases and Related Health Problems, Tenth Revision,* criteria. Each study also included a measure of depressive symptoms: the Beck Depression Inventory II (BDI-II),^24^ 9-item Patient Health Questionnaire,^25^ or the 12-item General Health Questionnaire (GHQ-12)^26^ (eTable 2 in the Supplement).

### Data Analysis

#### Primary Outcomes

Depressive symptoms at 3 to 4 months after baseline were gathered in 2 ways. First, the *z* score (standardized and mean centered) of the depressive symptom scores in each study was calculated. Second, percentage differences were calculated by using the logarithm of depression scale scores and exponentiating the coefficient for the socioeconomic indicator in each model.

#### Secondary Outcomes

Secondary outcomes included remission on the primary depression measure in each study at 3 to 4 months after baseline (eTable 2 in the Supplement). Depressive symptoms at 6 to 8 months after baseline were captured with the *z* score calculated using the mean and SD for the scores at 3 to 4 months; in this way, the outcomes could be comparable with those found using the 3- to 4-month outcome and the logarithm of scores at 6 to 8 months. Depressive symptoms at 9 to 12 months after baseline were recorded.

#### Prognostic Indicators Under Consideration

The socioeconomic factors recorded at baseline in at least 2 of the included studies were as follows:

- Employment status (Cohen κ = 9; n = 4864). Employed (including full-time and part-time employment), unemployed (job seekers and those unemployed owing to ill health), and not seeking employment (stay-at-home parent, students, and retirees).
- Financial strain (κ = 7; n = 3656). Doing okay financially, just about getting by, and struggling financially.
- Housing status (κ = 8; n = 4397). Homeowner (including those with a mortgage), tenant, and other (living with family or friends, homeless, or living in a hostel).
- Highest level of educational attainment (κ = 8; n = 3689). Bachelor’s degree or higher, diplomas including foundation degrees or A-levels (equivalent to a high school diploma), general certificate of secondary education (UK national examinations usually conducted at age 16 years), and other (qualifications below the level of the general certificate of secondary education or no formal qualifications).

#### Confounders

For each of the prognostic factors, we adjusted for depressive disorder characteristics (ie, severity of depressive symptoms, comorbid panic disorder, duration of depression and anxiety, and a history of antidepressant treatment).^4^ We then adjusted for potential confounders that were not systematically missing (ie, all studies collected data on them): age, sex, marital status,^6^ and employment status, except in models where employment status was the prognostic indicator. In sensitivity analyses, we adjusted for variables that were systematically missing in separate models starting with those factors available in most of the included studies. These were housing status (κ = 8), long-term physical health condition status (yes or no; κ = 8), level of educational attainment (κ = 8), financial strain (κ = 7), and social support (κ = 6).^7^

To give associations independent of treatment type, a single treatment variable was created with dummy categories for each of the randomized groups in each of the individual studies and adjusted for in all random-effects models. Missing data were imputed using multiple imputation with chained equations (eAppendix in the Supplement).

### Statistical Analysis

#### Primary Analyses

The association between each socioeconomic factor and each outcome was assessed in 4 separate models and adjusted for different sets of confounders, using a 2-stage approach with DerSimonian and Laird random effects. Stata software, version 16 (StataCorp), was used in these calculations. This approach is preferred to 1-stage approaches where the included studies have sufficient sample sizes, and complex modeling is not required as it reduces biases by separating within-study from between-study effects.^27,28^

The 4 models were run for financial strain and level of educational attainment as ordinal variables and run again as categorical variables. Employment and housing status were only analyzed as categorical variables. To do this, dummy variables were created to compare each category (eg, unemployed) with a reference category (eg, employed). Model 1 included each prognostic factor adjusted for random treatment allocation in each study; model 2 added depressive symptom severity, depressive duration, anxiety duration, history of antidepressant treatment, and comorbid panic disorder; model 3 added age, sex, and marital status; and model 4 added employment status.

#### Secondary and Sensitivity Analyses

Five sensitivity analyses modeled variables that were not available in all studies. The first was model 4 with the addition of housing status. The second was model 4 with the addition of housing status and long-term health condition status. The third was model 4 with the addition of housing status, long-term health condition status, and the highest level of educational attainment.

Variables that were systematically missing differed across studies; therefore, 2 further sensitivity analyses were performed: model 4 with the addition of financial strain and model 4 with the addition of financial strain and social support. For the *z* score and log outcomes, linear regression models were fitted, the outcome variables were approximately normally distributed, and robust CIs were used to account for overly influential data points. Logistic models were fitted for remission (eTables 13 and 20 in the Supplement). Heterogeneity was assessed using prediction intervals, and the percentage of variation across studies was assessed using the *I*^2^ statistic.^29^

Additional sensitivity analyses were planned if heterogeneity was considerable,^29^ either from inspection of the forest plots or if the *I*^2^ was 75% or greater. If study quality was low or risk of bias was high, we removed the study contributing most to the heterogeneity, low quality, or high risk of bias.

#### Risk of Bias

Risk of bias assessments were conducted using the Quality in Prognosis Studies (QUIPS) tool,^3^ and the quality of evidence for each prognostic indicator was assessed using the Grading of Recommendations, Assessment, Development and Evaluation (GRADE) framework.^30^ GRADE ratings were made for each prognostic factor within each included study, across each study as a whole, and for each prognostic factor across all included studies. Ratings of indirectness and publication bias were only considered applicable for the prognostic factors across all included studies, not within any individual study. Two reviewers (J.B., R.S.) independently conducted these assessments with disagreements resolved by consensus among 4 reviewers (J.E.J.B., R.S., G.L., and S.P.). Risk of bias information is available in eTable 4 in the Supplement, and quality ratings assessment is listed in eTable 5 in the Supplement.

## Results

This systematic review and meta-analysis of individual patient data identified 9 eligible studies that provided individual patient data for 4864 patients (mean [SD] age, 42.5 (14.0) years; 3279 women [67.4%]; 1583 men [32.6%]). All 9 RCTs met the inclusion criteria (Figure), and all were conducted in the UK. Individual patient data from all the participants formed the present data set (Table 1).^31,32,33,34,35,36,37,38,39^ Study quality was judged to be high, and overall risk of bias was low, although study attrition was rated as high in 1 study^34^ and moderate in 3 others (eTables 4 and 5 in the Supplement).^31,36,38^ There was near-perfect agreement between the reviewers (interrater reliability: QUIPS, κ = 0.96; GRADE, κ = 1.00). Descriptive statistics are listed in Table 2 and eTable 8 in the Supplement.

**Figure.**
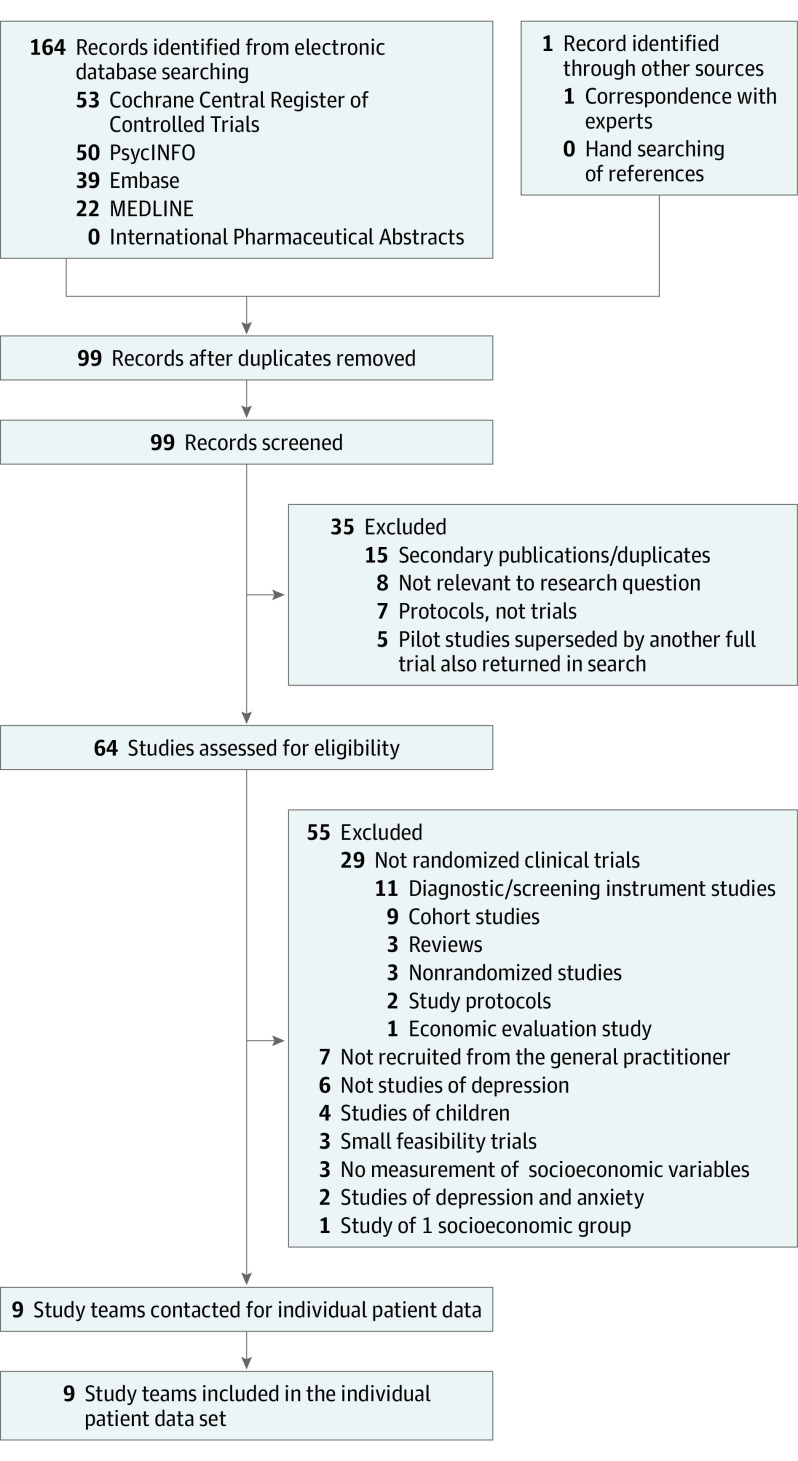
Flow of Studies Through Selection Process for Individual Patient Data Meta-analysis

**Table 1.** Description of Included Studies

Study, source	Patients, No.	Inclusion criteria	Employment status, %: employed; single, unemployed; not seeking employment	Financial strain, %: doing okay; just about getting by; struggling financially	Housing status, %: homeowner; tenant; other^a^	Highest level of educational attainment, %: bachelor’s degree or above; A-level^b^; GCSE; no formal qualifications	Baseline depressive severity, mean (SD)	Interventions	Outcome measure and primary postbaseline end point, primary measure (additional)
CADET, Richards et al,^31^ 2013	527	Adults ≥18 y, *ICD-10* depressive episode	45.2; 32.1; 22.8	NA	41.0; 48.6; 10.4	18.6; 27.9; 24.5; 29.0	PHQ-9, 17.7 (5.1)	Collaborative care vs TAU	PHQ-9 at 4 mo
COBALT, Wiles et al,^32^ 2013	469	Adults aged 18-75 y with treatment-resistant depression, scored ≥14 points on BDI-II	43.9; 32.2; 23.9	35.6; 37.1; 27.3	NA	20.5; 26.3; 28.1; 25.1	BDI-II, 31.8 (10.7)	CBT + TAU vs TAU	BDI-II (PHQ-9) at 3 mo
GENPOD, Wiles et al,^33^ 2012	601	Adults aged 18-74 y with depressive episode	59.4; 20.5; 20.1	35.1; 29.4; 35.6	48.1; 38.6; 13.3	25.3; 19.5; 41.4; 13.8	BDI-II, 33.7 (9.7)	Citalopram vs reboxetine	BDI-II at 3 mo
IPCRESS, Kessler et al,^34^ 2009	295	Adults scoring ≥14 points on BDI-II and clinician-confirmed diagnosis of depression	60.3; 11.9; 27.8	43.4; 28.5; 28.1	40.7; 42.4; 16.9	34.6; 29.8; 21.0; 14.6	BDI-II, 33.2 (8.8)	iCBT + TAU vs TAU + waiting list for iCBT	BDI-II at 4 mo
ITAS, Thomas et al,^35^ 2004	798	Adults aged ≥16 y, scored ≥12 points on CIS-R	52.0; 15.8; 32.3	44.7; 32.5; 22.9	62.0; 30.0; 8.1	NA	GHQ-12, 7.7 (3.2)	Recommendation + TAU vs TAU	GHQ-12 at 6 mo
MIR, Kessler et al,^36^ 2018	480	Adults ≥18 taking SSRIs or SNRIs at adequate dose for ≥6 wk, and scored ≥14 points on BDI-II	49.3; 21.3; 29.4	41.7; 31.7; 26.7	53.1; 38.8; 8.1	19.8; 28.1; 31.3; 20.8	BDI-II, 31.1 (9.9)	SSRI (SNRI) + mirtazapine vs SSRI (SNRI) + placebo	BDI-II (PHQ-9) at 3 mo
PANDA, Lewis et al,^37^ 2019	652	Adults presenting with low mood or depression to clinician in last 2 y, free of ADM for 8 wk up to baseline	66.4; 11.2; 22.4	55.8; 31.3; 12.9	40.0; 40.2; 19.8	35.3; 33.7; 22.2; 8.7	BDI-II, 23.9 (10.3)	Sertraline vs placebo	PHQ-9 (BDI-II) at 3 mo
REEACT, Gilbody et al,^38^ 2015	685	Adults with PHQ-9 ≥10 points presenting to clinician with depression	61.8; 18.0; 20.2	NA	50.6; 34.5; 14.9	39.2; 13.8; 46.1; 0.9	PHQ-9, 16.7 (4.3)	Moodgym vs Beating the Blues vs TAU^c^	PHQ-9 at 4 mo
TREAD, Chalder et al,^39^ 2012	361	Adults aged 18-69 y who met diagnostic criteria for MDD and scored ≥14 points on BDI-II	63.7; 13.3; 23.0	31.9; 34.4; 33.6	47.4; 39.6; 13.0	24.1; 28.8; 28.3; 18.8	BDI-II, 32.1 (9.2)	Physical activity + TAU vs TAU	BDI-II at 4 mo

Abbreviations: ADM, antidepressant medication; BDI-II, Beck Depression Inventory II; CADET, Clinical and Cost Effectiveness of Collaborative Care for Depression in UK Primary Care Trial; CIS-R, Clinical Interview Schedule Revised; COBALT, Cognitive Behavioural Therapy as an Adjunct to Pharmacotherapy for Treatment-Resistant Depression in Primary Care; GCSE, general certificate of secondary education; GENPOD, Genetic and Clinical Predictors of Treatment Response in Depression; GHQ-12, 12-item General Health Questionnaire; iCBT, internet-based therapist-delivered cognitive behavioral therapy; *ICD-10*, *International Statistical Classification of Diseases and Related Health Problems, Tenth Revision*; IPCRESS, Therapist-Delivered Internet Psychotherapy for Depression in Primary Care; ITAS, Computerised Patient-Specific Guidelines for Management of Common Mental Disorders in Primary Care; MDD, major depressive disorder; MIR, Mirtazapine Added to SSRIs or SNRIs for Treatment-Resistant Depression in Primary Care; NA, not available; PANDA, Clinical Effectiveness of Sertraline in Primary Care and the Role of Depression Severity and Duration; PHQ-9, 9-item Patient Health Questionnaire; REEACT, Computerised Cognitive Behaviour Therapy as Treatment for Depression in Primary Care; SNRI, serotonin and norepinephrine reuptake inhibitor; SSRI, selective serotonin reuptake inhibitor; TAU, treatment as usual; TREAD, Treating Depression With Physical Activity Trial.

^a^ Other includes living with family or friends, homeless, or living in a hostel.

^b^ A-level is an advanced level qualification that is equivalent to a high school diploma.

^c^ Types of cognitive behavioral therapy interventions.

**Table 2.** Baseline Characteristics of Overall Sample Across the 9 Included Studies, Using Observed Data

Self-reported baseline characteristic	No. (%)
Total sample size, No.	4864
Employment status	
Employed	2713 (55.8)
Not seeking employment	1199 (24.7)
Unemployed	949 (19.5)
Missing	3 (0.2)
Housing status	
Homeowner	2148 (48.9)
Tenant	1677 (38.2)
Other^a^	566 (12.9)
Missing	473 (9.7)
Financial strain	
Doing okay financially	1537 (42.1)
Just about getting by	1171 (32.1)
Struggling financially	939 (25.8)
Missing	1217 (25.0)
Highest level of educational attainment	
Bachelor’s degree or above	959 (28.0)
A-level of diplomas	905 (26.4)
GCSE	1016 (29.7)
None or other	543 (15.9)
Missing	1441 (29.6)
Age	4864 (100)
Mean (SD), y	42.45 (14.0)
Sex	
Female	3279 (67.4)
Male	1583 (32.6)
Missing	2 (0)
Marital status	
Married/cohabiting	2412 (49.6)
Single	1477 (30.7)
No longer married	975 (20.1)
Long-term physical health condition	
No	3244 (73.8)
Yes	1151 (26.2)
Missing	469 (9.6)
Social Support Scale score	2858 (58.8)
Mean (SD)	20.25 (3.9)
Past antidepressant use	
No	1241 (25.5)
Yes	3620 (74.5)
CIS-R durations^b^	4864 (100)
Depression	
Mean (SD)	3.32 (1.4)
Mean anxiety duration	4813 (99.0)
Mean (SD)	2.05 (1.0)
Comorbid panic disorder	
No	4439 (91.3)
Yes	425 (8.7)
Baseline BDI-II score	2858 (58.8)
Mean (SD)	30.44 (10.5)
Baseline PHQ-9 score	2812 (57.8)
Mean (SD)	15.71 (5.7)
Baseline GHQ-12 score	795 (16.3)
Mean (SD)	7.69 (3.2)
Attrition at 3-4 mo	
No	3411 (70.1)
Yes	658 (13.5)
NA	795 (16.3)
BDI-II score at 3-4 mo	1918 (39.4)
Mean (SD)	16.07 (12.0)
PHQ-9 score at 3-4 mo	2393 (49.2)
Mean (SD)	10.28 (6.7)
Remission at 3-4 mo	
No	1928 (56.6)
Yes	1480 (43.4)
BDI-II score at 6-8 mo	1236 (25.4)
Mean (SD)	18.64 (13.4)
PHQ-9 score at 6-8 mo	814 (16.7)
Mean (SD)	10.33 (6.8)
GHQ-12 score at 6-8 mo	585 (12.0)
Mean (SD)	3.80 (4.1)
Attrition at 6-8 mo	
No	1236 (25.4)
Yes	369 (7.6)
NA	3259 (67.0)
BDI-II score at 9-12 mo	1028 (21.1)
Mean (SD)	16.78 (12.9)
PHQ-9 score at 9-12 mo	1764 (32.3)
Mean (SD)	9.51 (6.7)
Attrition at 9-12 mo	
No	2005 (41.2)
Yes	516 (10.6)
NA	2343 (48.2)

Abbreviations: BDI-II, Beck Depression Inventory II; CIS-R, Clinical Interview Schedule Revised; GCSE, general certificate of secondary education; GHQ-12, 12-item General Health Questionnaire; NA, not applicable; PHQ-9, 9-item Patient Health Questionnaire.

^a^ Other includes living with family or friends, homeless, or living in a hostel.

^b^ Duration items are measured in 5 categories: (1) less than 2 weeks; (2) between 2 weeks and 6 months; (3) between 6 months and 1 year; (4) between 1 and 2 years; and (5) more than 2 years (eTable 2 in the Supplement).

### Associations Between Employment Status and Prognosis

Depressive symptom scores at 3 to 4 months were 47.3% (95% CI, 38.4%-56.8%) higher for unemployed patients than for employed patients, independent of treatment (Tables 3 and 4). Associations were lower in magnitude when adjusting for depressive disorder characteristics and when additionally adjusting for demographic variables (27.6%; 95% CI, 19.6%-36.1%). There were similar patterns of results adjusting for the systematically missing sociodemographic characteristics (eTable 15 in the Supplement) and at 6 to 8 months and 9 to 12 months (eTables 16, 17, 18, and 19 in the Supplement).

**Table 3.** Difference in *z* Score of Depressive Symptoms at 3 to 4 Months After Baseline per Unit Increase in Baseline Prognostic Indicator

Baseline variable^a^	Model 1^b^			Model 2^b^			Model 3^b^			Model 4^b^		
	*z* Score of depressive symptoms, mean difference (95% CI)	Studies, κ	*I*^2^, %	*z* Score of depressive symptoms, mean difference (95% CI)	Studies, κ	*I*^2^, %	*z* Score of depressive symptoms, mean difference (95% CI)	Studies, κ	*I*^2^, %	*z* Score of depressive symptoms, mean difference (95% CI)	Studies, κ	*I*^2^, %
Employment status												
Employed	0 [Reference]	NA	NA	0 [Reference]	NA	NA	0 [Reference]	NA	NA	0 [Reference]	NA	NA
Not seeking employment	0.09 (0.01 to 0.17)	8	12	0.07 (−0.01 to 0.14)	8	0	0.06 (−0.01 to 0.14)	8	0	NA	NA	NA
Unemployed	0.56 (0.44 to 0.68)	8	40	0.35 (0.24 to 0.46)	8	41	0.33 (0.22 to 0.44)	8	34	NA	NA	NA
Financial status												
Financial strain (ordinal)	0.22 (0.16 to 0.28)	6	24	0.09 (0.04 to 014)	6	0	0.08 (0.04 to 0.13)	6	0	0.06 (0.01 to 0.11)	6	0
Doing okay financially	0 [Reference]	NA	NA	0 [Reference]	NA	NA	0 [Reference]	NA	NA	0 [Reference]	NA	NA
Just about getting by	0.21 (0.09 to 0.32)	6	38	0.07 (−0.01 to 0.15)	6	0	0.08 (−0.01 to 0.16)	6	0	0.05 (−0.04 to 0.13)	6	0
Struggling financially	0.43 (0.32 to 0.54)	6	13	0.19 (0.09 to 0.29)	6	0	0.20 (0.10 to 0.30)	6	0	0.12 (0.02 to 0.22)	6	0
Housing status												
Homeowner	0 [Reference]	NA	NA	0 [Reference]	NA	NA	0 [Reference]	NA	NA	0 [Reference]	NA	NA
Tenant	0.32 (0.24 to 0.39)	7	0	0.15 (0.09 to 0.22)	7	0	0.16 (0.08 to 0.24)	7	0	0.12 (0.04 to 0.20)	7	0
Other housing status	0.38 (0.20 to 0.55)	7	59	0.24 (0.10 to 0.37)	7	40	0.26 (0.14 to 0.38)	7	0	0.21 (0.08 to 0.33)	7	0
Education												
Educational attainment (ordinal)	0.12 (0.06 to 0.17)	7	58	0.07 (0.03 to 0.12)	7	46	0.08 (0.03 to 0.12)	7	34	0.05 (0.02 to 0.09)	7	3
Bachelor’s degree or above	0 [Reference]	NA	NA	0 [Reference]	NA	NA	0 [Reference]	NA	NA	0 [Reference]	NA	NA
A-level	0.12 (0.03 to 0.22)	7	4	0.02 (−0.07 to 0.10)	7	0	0.01 (−0.08 to 0.09)	7	0	−0.01 (−0.10 to 0.07)	7	0
GCSE	0.20 (0.07 to 0.33)	7	48	0.10 (0.02 to 0.19)	7	0	0.10 (0.02 to 0.19)	7	0	0.06 (−0.02 to 0.15)	7	0
No formal qualifications	0.39 (0.20 to 0.58)	7	53	0.22 (0.09 to 0.36)	7	21	0.25 (0.13 to 0.36)	7	0	0.16 (0.05 to 0.28)	7	0

Abbreviations: GCSE, general certificate of secondary education; NA, not applicable.

^a^ Association for ordinal variables is per category increase from first category shown below the variable down to the last (eg, doing okay financially, to just about getting by, to struggling financially). Disorder characteristics were adjusted for baseline depressive symptom severity, average anxiety duration, depression duration, comorbid panic disorder, and a history of antidepressant treatment.

^b^ Model 1 included each prognostic factor adjusted for random treatment allocation in each study; model 2 included all model 1 variables and added depressive symptom severity, depressive duration, anxiety duration, history of antidepressant treatment, and comorbid panic disorder; model 3 included all model 2 variables and added age, sex, and marital status; and model 4 included all model 3 variables and added employment status.

**Table 4.** Percentage Difference in Depressive Symptom Scale Scores at 3 to 4 Months After Baseline per Unit Increase in Baseline Prognostic Indicator

Baseline variable^a^	Model 1^b^			Model 2^b^			Model 3^b^			Model 4^b^		
	Difference in depressive symptoms, % (95% CI)	Studies, κ	*I*^2^, %	Difference in depressive symptoms, % (95% CI)	Studies, κ	*I*^2^, %	Difference in depressive symptoms, % (95% CI)	Studies, κ	*I*^2^, %	Difference in depressive symptoms, % (95% CI)	Studies, κ	*I*^2^, %
Employment status												
Employed	0 [Reference]	NA	NA	0 [Reference]	NA	NA	0 [Reference]	NA	NA	0 [Reference]	NA	NA
Not seeking employment	10.6 (4.2 to 17.5)	8	0	8.8 (2.7 to 15.2)	8	0	8.9 (2.6 to 15.7)	8	0	NA	NA	NA
Unemployed	47.3 (38.4 to 56.8)	8	0	29.4 (21.7 to 37.6)	8	0	27.6 (19.6 to 36.1)	8	1	NA	NA	NA
Financial status												
Financial strain (ordinal)	14.7 (9.1 to 20.6)	6	40	5.4 (1.5 to 9.4)	6	0	4.6 (0.5 to 8.7)	6	0	2.5 (−1.5 to 6.6)	6	0
Doing okay financially	0 [Reference]	NA	NA	0 [Reference]	NA	NA	0 [Reference]	NA	NA	0 [Reference]	NA	NA
Just about getting by	14.8 (6.4 to 24.0)	6	12	4.9 (−2.0 to 12.2)	6	0	3.9 (−3.1 to 11.3)	6	0	2.8 (−4.1 to 10.1)	6	0
Struggling financially	30.2 (18.5 to 43.2)	6	30	11.3 (3.1 to 20.1)	6	0	9.8 (1.3 to 18.9)	6	0	5.2 (−3.0 to 14.1)	6	0
Housing status												
Homeowner	0 [Reference]	NA	NA	0 [Reference]	NA	NA	0 [Reference]	NA	NA	0 [Reference]	NA	NA
Tenant	25.8 (18.1 to 34.0)	7	0	12.8 (6.2 to 19.9)	7	0	13.4 (5.7 to 21.7)	7	9	9.5 (2.2 to 17.4)	7	0
Other housing status	35.0 (16.9 to 56.0)	7	63	22.2 (9.0 to 37.1)	7	45	22.5 (10.9 to 35.2)	7	0	17.6 (6.4 to 30.0)	7	0
Education												
Educational attainment (ordinal)	6.5 (2.1 to 11.1)	7	58	3.5 (−0.5 to 7.6)	7	55	3.7 (0.1 to 7.5)	7	44	1.0 (−3.2 to 5.5)	7	0
Bachelor’s degree or above	0 [Reference]	NA	NA	0 [Reference]	NA	NA	0 [Reference]	NA	NA	0 [Reference]	NA	NA
A-level	9.6 (1.9 to 17.9)	7	0	2.5 (−4.3 to 9.9)	7	0	1.8 (−5.1 to 9.2)	7	0	−0.1 (−6.9 to 7.2)	7	0
GCSE	11.4 (−0.0 to 24.1)	7	51	4.6 (−3.9 to 13.8)	7	29	4.7 (−4.0 to 14.1)	7	32	1.5 (−6.6 to 10.3)	7	26
No formal qualifications	25.1 (9.4 to 42.9)	7	45	12.2 (0.9 to 24.9)	7	26	13.6 (4.1 to 23.9)	7	0	6.4 (−2.8 to 16.5)	7	0

Abbreviations: GCSE, general certificate of secondary education; NA, not applicable.

^a^ Association for ordinal variables is per category increase from first category shown below the variable down to the last (eg, doing okay financially, to just about getting by, to struggling financially). Disorder characteristics were adjusted for baseline depressive symptom severity, average anxiety duration, depression duration, comorbid panic disorder, and history of antidepressant treatment.

^b^ Model 1 included each prognostic factor adjusted for random treatment allocation in each study; model 2 included all model 1 variables and added depressive symptom severity, depressive duration, anxiety duration, history of antidepressant treatment, and comorbid panic disorder; model 3 included all model 2 variables and added age, sex, and marital status; and model 4 included all model 3 variables and added employment status.

### Associations Between Financial Strain and Prognosis

Struggling financially was associated with worse prognosis relative to doing okay financially (Tables 3 and 4) (30.2%; 95% CI, 18.5%-43.2%). Associations were less strong when adjusting for depressive disorder characteristics (11.3%; 95% CI, 3.1%-20.1%). Additionally adjusting for employment status attenuated the associations altogether (5.2%; 95% CI, −3.0% to 14.1%). The same pattern was found at 6 to 8 months (eTables 9 and 10 in the Supplement), but there was no evidence of an association between financial strain and prognosis at 9 to 12 months (eTables 11 and 12 in the Supplement).

### Associations Between Housing Status and Prognosis

Tenants and patients with other housing status had worse prognoses than homeowners at 3 to 4 months (tenants: 25.8%; 95% CI, 18.1%-34.0%; other housing status: 35.0%; 95% CI, 16.9%-56.0%). The associations were weaker when adjusted for depressive disorder characteristics (tenants: 12.8%; 95% CI, 6.2%-19.9%; other housing status: 22.2%; 95% CI, 9.0%-37.1%) and weaker again when adjusted for employment status (tenants: 9.5%; 95% CI, 2.2%-17.4%; other housing status: 17.6%; 95% CI, 6.4%-30.0%), but those with other housing status still had considerably worse prognoses. The percentage difference in depressive symptoms at 3 to 4 months was 17.6% (95% CI, 6.4%-30.0%) (Table 4). Adjusting for long-term health conditions, educational attainment did not attenuate the outcomes. However, adjusting for financial status and social support did attenuate the outcomes to the point that there was no evidence of tenants having worse prognoses than homeowners. The latter analyses removed 2 studies without data on those variables (eTables 14 and 15 in the Supplement). There were similar findings at 6 to 8 months and 9 to 12 months, although CIs were wider, and the magnitude of associations was higher for tenants than for those with other housing statuses (at 6-8 months) (eTables 16, 17, 18, and 19 in the Supplement).

### Associations Between Highest Level of Educational Attainment and Prognosis

Patients with educational attainment levels where they had not obtained a bachelor’s degree or higher had worse prognoses at 3 to 4 months than those with higher-education degrees, independent of treatment (6.5%; 95% CI, 2.1%-11.1%) per unit decrease in qualifications but not after adjusting for other prognostic factors (1.0%; 95% CI, −3.2% to 5.5%) (Tables 3 and 4). Using the *z* score outcome, those with no formal qualifications had marginally worse depressive symptom scores than those with at least a bachelor’s degree after adjusting for all available confounders (0.16%; 95% CI, 0.05%-0.28%), but such evidence was not found with the log outcome (6.4%; 95% CI, −2.8% to 16.5%) (Table 4). At 6 to 8 months and 9 to 12 months after baseline, there was no evidence of associations between any of the educational attainment variables and prognosis after adjusting for disorder characteristics and employment status (eTables 9, 10, 11, and 12 in the Supplement).

### Further Sensitivity Analyses

There was no evidence of considerable heterogeneity in the primary analyses. In the secondary analyses where fewer studies were available, removing the additional studies that contributed most to high heterogeneity did not substantively change the magnitude of the associations (eTable 21 in the Supplement).

## Discussion

This systematic review and meta-analysis found that unemployed patients had considerably worse depression treatment prognoses than employed patients. After adjusting for all other available prognostic variables, their depressive symptom scores were 28% (at 3-4 months), 30% (at 6-8 months), and 37% (at 9-12 months) higher than those of employed patients. In absolute terms, unemployed patients scored approximately 4 points higher at 3 to 4 months after baseline and 6 points higher at both 6 to 8 months and 9 to 12 months after baseline on the BDI-II than employed patients. In addition, compared with homeowners, depressive symptom scores were 18% higher for patients living with family or friends, in hostels, or homeless, which is equivalent to approximately 3 points on the BDI-II. These associations might be considered clinically important by exceeding some estimates for the minimal clinically important difference.^40^ Financial strain and educational attainment were associated with prognosis independent of treatment, but there was little evidence of associations after adjusting for depressive disorder characteristics and employment status.

### Strengths and Limitations

To our knowledge, this was the first systematic review and individual patient data set meta-analysis to consider the associations of socioeconomic factors with prognosis across different types of treatment. All 4864 participants from the eligible RCTs were included, bringing greater precision to estimates of these associations than in past studies. In contrast to past reviews, we selected studies that included adults with depression who sought treatment in primary care settings, a very common route into treatment internationally.^15,16^ This partly limited the number of studies found to meet inclusion criteria (6 studies were excluded for not recruiting in primary care) but had the advantage of ensuring that there was a minimum population for whom the findings may be generalizable. This offered an improvement on the extant literature in which there is little information about from where participants were recruited.

The patients studied here had all consented to participate in RCTs, and all the studies were conducted in the UK. Therefore, this may be a biased sample compared with all patients with depression and could further limit generalizability. However, 8 of 9 studies were pragmatic trials; therefore, the participants should be broadly representative of other patients with depression in primary care. It is important to emphasize that the associations with prognosis were averaged across a wide range of different treatments and are, in that sense, associated with prognosis irrespective of the treatment that was given. The findings should, therefore, be informative for clinicians assessing patients with depression before treatment is started.

Only studies that used the same assessment measure to determine diagnosis and assess baseline symptoms and depressive disorder characteristics confounders were included. This minimized bias in harmonizing the data across studies and ensured that data were available on the same confounders across all studies. This could have reduced the potential pool of studies, but no studies were excluded solely for not using the CIS-R. Many studies contained no comprehensive measure of anxiety disorder symptoms or diagnoses, and their inclusion would, therefore, not have allowed us to meet the aims of this study. Further, as individual patient data were available for all studies that met the inclusion criteria, a common source of selection bias that can occur when only a subset of eligible trials provide individual patient data was avoided.

Data were extracted, cleaned, and checked by multiple reviewers, adding robustness,^41^ although only a single reviewer assessed articles at the title and abstract stage, which potentially introduced additional bias. Adjustments were made for a number of potential confounders, but residual confounding cannot be ruled out. Further, it is possible that adjusting for baseline depressive severity may have led to underestimating the associations of the socioeconomic factors with prognosis, as these factors could mediate those associations. The same could be true of the models adjusted for employment status, which attenuated a number of otherwise potentially meaningful associations.

## Conclusions

Findings of this systematic review and meta-analysis of individual patient data suggest that patients in primary care with depression who were socioeconomically disadvantaged (ie, those who were unemployed, struggling financially, not homeowners, or had no formal educational qualifications) had poorer prognoses regardless of the type of treatment they received and the severity of depression. Our results highlight employment and housing status as being clinically important as the outcomes were larger than previous estimates of proportional minimal clinically important differences for patients with depression.^40^ These factors are easy to assess and doing so during the pretreatment phase could help to inform future management of depression. Interventions to support patients to gain or maintain employment, or to achieve stable housing, have been effective in improving quality of life, functioning, and depressive symptoms.^42,43^ Obtaining support for such problems may be as effective as more conventional treatments for depression, and addressing these needs may make it easier for patients to engage in and benefit from psychotherapy or pharmacotherapy for depression.^44^ This may be particularly important at present given concerns of increased vulnerability owing to the effect of the COVID-19 pandemic on employment and housing.^45^ Future studies may investigate the optimal order with which to offer support for employment or housing, as well as more intensive conventional treatment strategies, including longer follow-up, for the management of depression in adults that present with clinical indicators of poorer prognosis.^46^ At a public health level, knowing that socioeconomic disadvantage is associated with a worse depression treatment prognosis suggests that reducing socioeconomic inequalities may improve mental health through an association with treatment prognosis as well as a reduction in the incidence of mental health problems.^47,48^

## Notes

Supplement. **eTable 1.** Bibliographic Database Searches and Results **eAppendix.** PRISMA-IPD Checklist and Additional Details on Methods and Analysis **eTable 2. **Measures Used Across the RCTs Meeting Inclusion Criteria for the Present Study **eTable 3. **Ethical Approval and Trial Registration Details of the RCTs Meeting Inclusion Criteria for This Study **eTable 4.** QUIPS Risk of Bias Ratings **eTable 5.** GRADE Quality Rating of Evidence for Each Type Prognostic Factor Assessed **eTable 6.** Bibliographic Database Searches and Results for Preliminary Searches **eTable 7.** Results From Database Searches and Results for Preliminary Searches and Reasons for Exclusion **eTable 8.** Mean and SDs of the Overall *z *Score on the Depressive Symptom Measures at Baseline by Each Category of Each Socioeconomic Status Variable, Within Each Included Study **eTable 9.** Difference in *z *Score of Depressive Symptoms at 6-8 Months After Baseline Per Unit Increase in Baseline Prognostic Indicator **eTable 10.** Percentage Difference in Depressive Symptom Scale Scores at 6-8 Months After Baseline Per Unit Increase in Baseline Prognostic Indicator **eTable 11.** Difference in *z* Score of Depressive Symptoms at 9-12 Months After Baseline Per Unit Increase in Baseline Prognostic Indicator **eTable 12.** Percentage Difference in Depressive Symptom Scale Scores at 9-12 Months After Baseline Per Unit Increase in Baseline Prognostic Indicator **eTable 13.** Odds Ratios for Being in Remission at 3-4 Months After Baseline Per Unit Increase in Baseline Prognostic Indicator **eTable 14.** Difference in *z* Score of Depressive Symptoms at 3-4 Months After Baseline Per Unit Increase in Baseline Prognostic Indicator, Adjusting for Variables That Were Systematically Missing in Some Studies **eTable 15.** Percentage Difference in Depressive Symptom Scale Scores at 3-4 Months After Baseline Per Unit Increase in Baseline Prognostic Indicator Adjusting for Variables That Were Systematically Missing in Some Studies **eTable 16.** Difference in *z* Score of Depressive Symptom Scale Scores at 6-8 Months After Baseline Per Unit Increase in Baseline Prognostic Indicator Adjusting for Variables That Were Systematically Missing in Some Studies **eTable 17.** Percentage Difference in Depressive Symptom Scale Scores at 6-8 Months After Baseline Per Unit Increase in Baseline Prognostic Indicator Adjusting for Variables That Were Systematically Missing in Some Studies **eTable 18.** Difference in *z* Score of Depressive Symptoms at 9-12 Months After Baseline Per Unit Increase in Baseline Prognostic Indicator Adjusting for Variables That Were Systematically Missing in Some Studies **eTable 19.** Percentage Difference in Depressive Symptom Scale Scores at 9-12 Months After Baseline Per Unit Increase in Baseline Prognostic Indicator Adjusting for Variables That Were Systematically Missing in Some Studies **eTable 20.** Odds Ratios for Being in Remission at 3-4 Months After Baseline Per Unit Increase in Baseline Prognostic Indicator, Adjusting for Variables That Were Systematically Missing in Some Studies **eTable 21.** Results of Original Analyses and Corresponding Sensitivity Analyses Removing Studies Due to Heterogeneity
eReferences
