# Supplementary material for: Socioeconomic Indicators of Treatment Prognosis for Adults With Depression: A Systematic Review and Individual Patient Data Meta-analysis
Source: JAMA Psychiatry. 2022 Mar 9;79(5):406–16. doi: 10.1001/jamapsychiatry.2022.0100 (PMC8908224; doi:10.1001/jamapsychiatry.2022.0100)
Supplement: Supplement. — eTable 1. Bibliographic Database Searches and Results eAppendix. PRISMA-IPD Checklist and Additional Details on Methods and Analysis eTable 2. Measures Used Across the RCTs Meeting Inclusion Criteria for the Present Study eTable 3. Ethical Approval and Trial Registration Details of the RCTs Meeting Inclusion Criteria for This Study eTable 4. QUIPS Risk of Bias Ratings eTable 5. GRADE Quality Rating of Evidence for Each Type Prognostic Factor Assessed eTable 6. Bibliographic Database Searches and Results for Preliminary Searches eTable 7. Results From Database Searches and Results for Preliminary Searches and Reasons for Exclusion eTable 8. Mean and SDs of the Overall z Score on the Depressive Symptom Measures at Baseline by Each Category of Each Socioeconomic Status Variable, Within Each Included Study eTable 9. Difference in z Score of Depressive Symptoms at 6-8 Months After Baseline Per Unit Increase in Baseline Prognostic Indicator eTable 10. Percentage Difference in Depressive Symptom Scale Scores at 6-8 Months After Baseline Per Unit Increase in Baseline Prognostic Indicator eTable 11. Difference in z Score of Depressive Symptoms at 9-12 Months After Baseline Per Unit Increase in Baseline Prognostic Indicator eTable 12. Percentage Difference in Depressive Symptom Scale Scores at 9-12 Months After Baseline Per Unit Increase in Baseline Prognostic Indicator eTable 13. Odds Ratios for Being in Remission at 3-4 Months After Baseline Per Unit Increase in Baseline Prognostic Indicator eTable 14. Difference in z Score of Depressive Symptoms at 3-4 Months After Baseline Per Unit Increase in Baseline Prognostic Indicator, Adjusting for Variables That Were Systematically Missing in Some Studies eTable 15. Percentage Difference in Depressive Symptom Scale Scores at 3-4 Months After Baseline Per Unit Increase in Baseline Prognostic Indicator Adjusting for Variables That Were Systematically Missing in Some Studies eTable 16. Difference in z Score of Depressive Symptom Scale [file jamapsychiatry-e220100-s001.pdf]

Supplementary Online Content

Buckman JEJ, Saunders R, Stott J, et al. Socioeconomic indicators of treatment prognosis for adults with depression: a systematic review and individual patient data meta-analysis. *JAMA Psychiatry*. Published online March 9, 2022. doi:10.1001/jamapsychiatry.2022.0100

eTable 1. Bibliographic Database Searches and Results

eAppendix. PRISMA-IPD Checklist and Additional Details on Methods and Analysis

eTable 2. Measures Used Across the RCTs Meeting Inclusion Criteria for the Present Study

eTable 3. Ethical Approval and Trial Registration Details of the RCTs Meeting Inclusion Criteria for This Study

eTable 4. QUIPS Risk of Bias Ratings

eTable 5. GRADE Quality Rating of Evidence for Each Type Prognostic Factor Assessed

eTable 6. Bibliographic Database Searches and Results for Preliminary Searches

eTable 7. Results From Database Searches and Results for Preliminary Searches and Reasons for Exclusion

eTable 8. Mean and SDs of the Overall *z* Score on the Depressive Symptom Measures at Baseline by Each Category of Each Socioeconomic Status Variable, Within Each Included Study

eTable 9. Difference in *z* Score of Depressive Symptoms at 6-8 Months After Baseline Per Unit Increase in Baseline Prognostic Indicator

eTable 10. Percentage Difference in Depressive Symptom Scale Scores at 6-8 Months After Baseline Per Unit Increase in Baseline Prognostic Indicator

eTable 11. Difference in *z* Score of Depressive Symptoms at 9-12 Months After Baseline Per Unit Increase in Baseline Prognostic Indicator

eTable 12. Percentage Difference in Depressive Symptom Scale Scores at 9-12 Months After Baseline Per Unit Increase in Baseline Prognostic Indicator

eTable 13. Odds Ratios for Being in Remission at 3-4 Months After Baseline Per Unit Increase in Baseline Prognostic Indicator

eTable 14. Difference in *z* Score of Depressive Symptoms at 3-4 Months After Baseline Per Unit Increase in Baseline Prognostic Indicator, Adjusting for Variables That Were Systematically Missing in Some Studies

eTable 15. Percentage Difference in Depressive Symptom Scale Scores at 3-4 Months After Baseline Per Unit Increase in Baseline Prognostic Indicator Adjusting for Variables That Were Systematically Missing in Some Studies

© 2022 Buckman JEJ et al. *JAMA Psychiatry.*

eTable 16. Difference in *z* Score of Depressive Symptom Scale Scores at 6-8 Months After Baseline Per Unit Increase in Baseline Prognostic Indicator Adjusting for Variables That Were Systematically Missing in Some Studies

eTable 17. Percentage Difference in Depressive Symptom Scale Scores at 6-8 Months After Baseline Per Unit Increase in Baseline Prognostic Indicator Adjusting for Variables That Were Systematically Missing in Some Studies

eTable 18. Difference in *z* Score of Depressive Symptoms at 9-12 Months After Baseline Per Unit Increase in Baseline Prognostic Indicator Adjusting for Variables That Were Systematically Missing in Some Studies

eTable 19. Percentage Difference in Depressive Symptom Scale Scores at 9-12 Months After Baseline Per Unit Increase in Baseline Prognostic Indicator Adjusting for Variables That Were Systematically Missing in Some Studies

eTable 20. Odds Ratios for Being in Remission at 3-4 Months After Baseline Per Unit Increase in Baseline Prognostic Indicator, Adjusting for Variables That Were Systematically Missing in Some Studies

eTable 21. Results of Original Analyses and Corresponding Sensitivity Analyses Removing Studies Due to Heterogeneity

eReferences

This supplementary material has been provided by the authors to give readers additional information about their work.

Details of search terms and search results for RCTs to form IPD dataset

eTable 1. Bibliographic Database Searches and Results

Searches	Results
**Cochrane CENTRAL Trial Register (searched on 8th October 2021)**	
1. ("Depression" or "MDD" or "Unipolar" or "Depressive"):ti,ab,kw (Word variations have been searched)	87938
2. ("RCT" or "controlled trial" or "randomized controlled trial" or "clinical trial"):ti,ab,kw (Word variations have been searched)	1144748
3. ("CIS-R" or "Clinical Interview Schedule" or "Revised Clinical Interview Schedule" or "Clinical Interview Schedule Revised"):ti,ab,kw (Word variations have been searched)	67
4. #1 and #2 and #3	**53**
**Embase 1947 to 2021 October 07**	
1. (depression or Depressive disorder or Major depression or Unipolar depression or MDD).mp.	769534
2. exp controlled clinical trial/ or exp "randomized controlled trial (topic)"/ or exp "clinical trial"/	1859661
3. ("Clinical Interview Schedule" or "CIS-R" or "CISR" or "Revised clinical interview schedule" or "clinical interview schedule revised").af.	893
4. 1 and 2 and 3	**39**
**International Pharmaceutical Abstracts 1970 to September 2021**	
1. (depression or Depressive disorder or Major depression or Unipolar depression or MDD).mp.	10859
2. (RCT or controlled trial or randomized controlled trial or clinical trial).mp.	16472
3. ("Clinical Interview Schedule" or "CIS-R" or "CISR" or "Revised clinical interview schedule" or "clinical interview schedule revised").af.	4
4. 1 and 2 and 3	**0**
**Ovid MEDLINE 1946 to October 07, 2021**	
1. exp major depression/ or exp "depression (emotion)"/	132832
2. exp Depressive Disorder, Major/	33340
3. exp Depressive Disorder, Major/ or exp Depressive Disorder/ or exp Depression/	233903
4. 1 or 2 or 3	233903
5. exp controlled clinical trial/ or exp "randomized controlled trial (topic)"/	636694
6. ("Clinical Interview Schedule" or "CIS-R" or "CISR" or "Revised clinical interview schedule" or "clinical interview schedule revised").af.	651
7. 4 and 5 and 6	**22**
**PsycINFO 1806 to October Week 1 2021**	
1. exp major depression/ or exp "depression (emotion)"/	166578
2. (depression or Depressive disorder or Major depression or Unipolar depression or MDD).mp.	361622
3. 1 or 2	361850
4. exp "randomized controlled trial (topic)"/ or exp "clinical trial"/ or exp "controlled trial"/ or exp "randomized clinical trial"/	12990
5. (RCT or controlled trial or randomized controlled trial or clinical trial).mp.	47329
6. 4 or 5	54073
7. ("Clinical Interview Schedule" or "CIS-R" or "CISR" or "Revised clinical interview schedule" or "clinical interview schedule revised").af.	1275
8. 3 and 6 and 7	**50**

© 2022 Buckman JEJ et al. *JAMA Psychiatry.*

eAppendix. PRISMA-IPD Checklist and Additional Details on Methods and Analysis

PRISMA-IPD Checklist of items to include when reporting a systematic review and meta-analysis of individual participant data (IPD)

PRISMA-IPD Section/topic	Item No	Checklist item	Reported on page
**Title**			
Title	1	Identify the report as a systematic review and meta-analysis of individual participant data.	1
**Abstract**			
Structured summary	2	Provide a structured summary including as applicable:	3
		**Background**: state research question and main objectives, with information on participants, interventions, comparators and outcomes.	
		**Methods**: report eligibility criteria; data sources including dates of last bibliographic search or elicitation, noting that IPD were sought; methods of assessing risk of bias.	
		**Results**: provide number and type of studies and participants identified and number (%) obtained; summary effect estimates for main outcomes (benefits and harms) with confidence intervals and measures of statistical heterogeneity. Describe the direction and size of summary effects in terms meaningful to those who would put findings into practice.	
		**Discussion:** state main strengths and limitations of the evidence, general interpretation of the results and any important implications.	
		**Other:** report primary funding source, registration number and registry name for the systematic review and IPD meta-analysis.	
**Introduction**			
Rationale	3	Describe the rationale for the review in the context of what is already known.	2-4
Objectives	4	Provide an explicit statement of the questions being addressed with reference, as applicable, to participants, interventions, comparisons, outcomes and study design (PICOS). Include any hypotheses that relate to particular types of participant-level subgroups.	4
**Methods**			
Protocol and registration	5	Indicate if a protocol exists and where it can be accessed. If available, provide registration information including registration number and registry name. Provide publication details, if applicable.	3,5

© 2022 Buckman JEJ et al. *JAMA Psychiatry.*

Eligibility criteria	6	Specify inclusion and exclusion criteria including those relating to participants, interventions, comparisons, outcomes, study design and characteristics (e.g. years when conducted, required minimum follow-up). Note whether these were applied at the study or individual level i.e. whether eligible participants were included (and ineligible participants excluded) from a study that included a wider population than specified by the review inclusion criteria. The rationale for criteria should be stated.	5
Identifying studies - information sources	7	Describe all methods of identifying published and unpublished studies including, as applicable: which bibliographic databases were searched with dates of coverage; details of any hand searching including of conference proceedings; use of study registers and agency or company databases; contact with the original research team and experts in the field; open adverts and surveys. Give the date of last search or elicitation.	5
Identifying studies - search	8	Present the full electronic search strategy for at least one database, including any limits used, such that it could be repeated.	S Tab 1
Study selection processes	9	State the process for determining which studies were eligible for inclusion.	5
Data collection processes	10	Describe how IPD were requested, collected and managed, including any processes for querying and confirming data with investigators. If IPD were not sought from any eligible study, the reason for this should be stated (for each such study).	S Tab 3
		If applicable, describe how any studies for which IPD were not available were dealt with. This should include whether, how and what aggregate data were sought or extracted from study reports and publications (such as extracting data independently in duplicate) and any processes for obtaining and confirming these data with investigators.	N/A
Data items	11	Describe how the information and variables to be collected were chosen. List and define all study level and participant level data that were sought, including baseline and follow-up information. If applicable, describe methods of standardising or translating variables within the IPD datasets to ensure common scales or measurements across studies.	6,7, S Tab 3
IPD integrity	A1	Describe what aspects of IPD were subject to data checking (such as sequence generation, data consistency and completeness, baseline imbalance) and how this was done.	3, 8, S Tab 3
Risk of bias assessment in individual studies.	12	Describe methods used to assess risk of bias in the individual studies and whether this was applied separately for each outcome. If applicable, describe how findings of IPD checking were used to inform the assessment. Report if and how risk of bias assessment was used in any data synthesis.	3,9
Specification of outcomes and effect measures	13	State all treatment comparisons of interests. State all outcomes addressed and define them in detail. State whether they were pre-specified for the review and, if applicable, whether they were primary/main or secondary/additional outcomes. Give the principal measures of effect (such as risk ratio, hazard ratio, difference in means) used for each outcome.	6

© 2022 Buckman JEJ et al. *JAMA Psychiatry.*

Synthesis methods	14	Describe the meta-analysis methods used to synthesise IPD. Specify any statistical methods and models used. Issues should include (but are not restricted to):<br>• Use of a one-stage or two-stage approach.<br>• How effect estimates were generated separately within each study and combined across studies (where applicable).<br>• Specification of one-stage models (where applicable) including how clustering of patients within studies was accounted for.<br>• Use of fixed or random effects models and any other model assumptions, such as proportional hazards.<br>• How (summary) survival curves were generated (where applicable).<br>• Methods for quantifying statistical heterogeneity (such as I^2 and τ^2).<br>• How studies providing IPD and not providing IPD were analysed together (where applicable).<br>• How missing data within the IPD were dealt with (where applicable).	8

Exploration of variation in effects	A2	If applicable, describe any methods used to explore variation in effects by study or participant level characteristics (such as estimation of interactions between effect and covariates). State all participant-level characteristics that were analysed as potential effect modifiers, and whether these were pre-specified.	6-8
Risk of bias across studies	15	Specify any assessment of risk of bias relating to the accumulated body of evidence, including any pertaining to not obtaining IPD for particular studies, outcomes or other variables.	9
Additional analyses	16	Describe methods of any additional analyses, including sensitivity analyses. State which of these were pre-specified.	9
**Results**			
Study selection and IPD obtained	17	Give numbers of studies screened, assessed for eligibility, and included in the systematic review with reasons for exclusions at each stage. Indicate the number of studies and participants for which IPD were sought and for which IPD were obtained. For those studies where IPD were not available, give the numbers of studies and participants for which aggregate data were available. Report reasons for non-availability of IPD. Include a flow diagram.	Figure 1
Study characteristics	18	For each study, present information on key study and participant characteristics (such as description of interventions, numbers of participants, demographic data, unavailability of outcomes, funding source, and if applicable duration of follow-up). Provide (main) citations for each study. Where applicable, also report similar study characteristics for any studies not providing IPD.	Table 1
IPD integrity	A3	Report any important issues identified in checking IPD or state that there were none.	3, S Tab 3
Risk of bias within studies	19	Present data on risk of bias assessments. If applicable, describe whether data checking led to the up-weighting or down-weighting of these assessments. Consider how any potential bias impacts on the robustness of meta-analysis conclusions.	9, S Tab 4-5

© 2022 Buckman JEJ et al. *JAMA Psychiatry.*

Results of individual studies	20	For each comparison and for each main outcome (benefit or harm), for each individual study report the number of eligible participants for which data were obtained and show simple summary data for each intervention group (including, where applicable, the number of events), effect estimates and confidence intervals. These may be tabulated or included on a forest plot.	Tab 3-4, 8-19
Results of syntheses	21	Present summary effects for each meta-analysis undertaken, including confidence intervals and measures of statistical heterogeneity. State whether the analysis was pre-specified, and report the numbers of studies and participants and, where applicable, the number of events on which it is based.	NA, 2,3,10, 11, Tab 4, S Tab 3-4, 8-19
		When exploring variation in effects due to patient or study characteristics, present summary interaction estimates for each characteristic examined, including confidence intervals and measures of statistical heterogeneity. State whether the analysis was pre-specified. State whether any interaction is consistent across trials.	
		Provide a description of the direction and size of effect in terms meaningful to those who would put findings into practice.	
Risk of bias across studies	22	Present results of any assessment of risk of bias relating to the accumulated body of evidence, including any pertaining to the availability and representativeness of available studies, outcomes or other variables.	9, S Tab
Additional analyses	23	Give results of any additional analyses (e.g. sensitivity analyses). If applicable, this should also include any analyses that incorporate aggregate data for studies that do not have IPD. If applicable, summarise the main meta-analysis results following the inclusion or exclusion of studies for which IPD were not available.	11, S Tab 20
**Discussion**			
Summary of evidence	24	Summarise the main findings, including the strength of evidence for each main outcome.	2,3,10
Strengths and limitations	25	Discuss any important strengths and limitations of the evidence including the benefits of access to IPD and any limitations arising from IPD that were not available.	11-12
Conclusions	26	Provide a general interpretation of the findings in the context of other evidence.	12-13
Implications	A4	Consider relevance to key groups (such as policy makers, service providers and service users). Consider implications for future research.	2,10
**Funding**			
Funding	27	Describe sources of funding and other support (such as supply of IPD), and the role in the systematic review of those providing such support.	14

© 2022 Buckman JEJ et al. *JAMA Psychiatry.*

A1 – A3 denote new items that are additional to standard PRISMA items. A4 has been created as a result of re-arranging content of the standard PRISMA statement to suit the way that systematic review IPD meta-analyses are reported.

© Reproduced with permission of the PRISMA IPD Group, which encourages sharing and reuse for non-commercial purposes

Additional Information on the Prognostic indicators

1) Employment Status (available in 9 studies, n=4864) – An eight category variable capturing this existed in the dataset: i) employed full-time; ii) employed part-time; iii) houseperson; iv) retired; v) student; vi) unemployed job seeker; vii) unemployed due to ill health; viii) other. However, based on previous work with the data which included the studies included here, there were insufficient numbers of participants in some categories in some studies to use all eight categories above. For example, there was only one houseperson in the REEACT study, five in the MIR study and nine in the COBALT study. There were also only four retirees in the IPCRESS study, and seven in the TREAD study. So, these categories were collapsed together to form a three category variable: i) employed (including full time and part time employed); ii) unemployed (including unemployed job seeker and unemployed due to ill health); and iii) not seeking employment (including all other categories).
2) Financial Strain (available in seven studies, n=3656) – A five category variable capturing this existed in the data provided: i) Living comfortably; ii) Doing alright financially; iii) Just about getting by; iv) finding it difficult to make ends meet; v) very difficult to make ends meet. Again, there were low numbers in some categories within some of the studies, so this variable was collapsed into three categories: i) Doing OK Financially (living comfortably or doing alright financially); ii) just about getting by; iii) struggling financially (difficult or very difficult to make ends meet).
3) Housing Status (available in eight studies, n=4397) – a six category variable capturing housing tenancy status existed in the data: i) Homeowner, ii) Tenant, iii) Living with family/friends; iv) Hostel, v) Homeless, vi) Other. There were very few participants that reported being homeless or living in hostels in most of the studies, so this variable will be re-categorised into three categories: i) Homeowner; ii) Tenant; and iii) Other.
4) Highest Level of Educational Attainment (available in eight studies, n=3689) – a six category variable capturing this existed: i) Degree of above; ii) Foundation degree, higher national diploma, or equivalent; iii) A-level; iv) GCSE; v) Other qualifications (below the level of GCSEs); vi) No formal qualifications. There were no participants with "Other qualifications" in COBALT, and none with "No formal qualifications" in REEACT and MIR. This variable was therefore re-categorised into the following four categories: i) Bachelor's Degree or higher; ii) A-levels or Diplomas including Foundation Degrees; iii) GCSE; iv) Other qualifications (below the level of GCSEs) or no formal qualifications.

Additional Information on Calculation of 'Percentage Difference' primary outcome:

To calculate the 'percentage difference' primary outcome, a new variable was created by calculating the natural logarithm of the depressive symptom measure scores for each participant at 3-4 months post-baseline, irrespective of the depressive measure used (i.e. the variable contained the natural logarithm of scores for participants that had completed the PHQ-9 and those that had completed the BDI-II). This variable was fitted as a continuous outcome variable in the regression models, and the exponent (e^x) of the coefficient for the prognostic variable (the socioeconomic variable) was then calculated to give the effect estimate per one-unit increase in that variable for the ordinal socioeconomic variables or comparing the stated category to the reference category (e.g. employed compared to unemployed). 95% confidence intervals were created with the exponentiated values. The percentage difference for the socioeconomic variable in each model was calculated as: 100*(the exponentiated coefficient -1). These percentage differences could then be compared with estimates for minimal clinically important differences on the PHQ-9 and BDI-II from previous research with primary care patients.[1]

Data Extraction

Raw data were extracted for each study participant of all studies meeting inclusion criteria that agreed to provide individual patient data. Data were cleaned one study at a time, independently by two reviewers (JB and RS).

© 2022 Buckman JEJ et al. *JAMA Psychiatry.*

Data Integrity Checks

Integrity of all baseline and endpoint data for each study were checked with the study team and against details published about each study. The numbers of participants included in this dataset for four studies was slightly different from those in the published articles about the individual studies. This is because a very small number of cases were removed as they had missing data on over 75% of the variables at baseline, this resulted in two patients being removed from the IPCRESS study and one from the PANDA study. For the CADET study 54 participants withdrew after the study was completed so their data were not made available, and for the ITAS study there were complete data for 36 more participants than reported in the publications about that study.

Missing Data

Missing data were imputed using multiple imputation with chained equations (MICE) in Stata 16.0. Data not reasonably able to be log transformed to meet normality assumptions, were imputed using predictive mean matching (PMM) via a k-nearest neighbours approach as it is considered to be more appropriate for non-normal continuous variables [2], here we used k=10. Linear regression was used for approximately normally distributed continuous variables, logistic regression models for binary variables, and ordinal and multinomial regression models for ordered and unordered categorical variables respectively. All imputation models were built using data on baseline and outcome variables following conventions [3]. Only variables with less than 50% missing data were imputed. All imputation models were run to produce 50 imputed datasets.

Software & Packages

Stata SE 16:[4] ipdmetan,[5] MICE,[6] mi impute pmm[7] packages.

Patient and Public Involvement

Service user advisory groups of two primary care mental health services in central London and the expert service user researchers of the Service User Research Forum (SURF) were consulted for advice on the design, conduct, and dissemination of this study.

Details of preliminary searches, and additions, deviations and changes to protocols

We registered the process of finding studies and the research questions for this study on PROSPERO (CRD42019129512) and produced a protocol paper which was amended twice. Below we explain the amendments made and the process of finding studies and forming the dataset for this study.

We started this project by running some preliminary searches informed by consultation with a librarian at University College London, to identify studies of depression in primary care using the MEDLINE database via OVID, hand-searching through reference lists of existing systematic reviews and contacting a number of experts to enquire about unpublished or ongoing studies. No limits or filters were applied to the searches and no automatic updates were applied, although searches were re-run as detailed below. In these searches we found that the Clinical Interview Schedule, Revised version (CIS-R)[8] was the most commonly used comprehensive measures of depressive and anxiety symptoms and disorders in RCTs of depression set in primary care, among studies returned in the searches. Ten studies used the CIS-R at baseline to determine diagnosis (seven published and three protocols for studies that would soon be completed). Only one of the returned studies used the Schedules for Clinical Assessment in Neuropsychiatry (SCAN)[9] but did not meet all other inclusion criteria as no details were given on any socioeconomic factors and their associations with prognostic outcomes.[10] In addition, one study used the full Structured Clinical Interview for DSM (SCID)[11] but it too did not provide information on any socioeconomic factors measured at baseline,[12] see Supplementary Tables 6 and 7 for details. We therefore refined our preliminary searches to look for studies that used the CIS-R, and the use of CIS-R at baseline was made an inclusion criterion to minimize biases when harmonising data,[13] and ensure included studies had data on the depressive 'disorder characteristics' found to be independently associated with prognosis,[14] to meet the aim of ascertaining whether socioeconomic factors can add to prognostic information, in addition to routinely assessed clinical factors.

One of the senior investigators involved in this project (GL) was a lead or co-investigator on a number of trials that used the CIS-R and we made contact with the chief investigators of those studies to ask for in-principle agreement to access IPD from their trials. We then applied for funding for this project. Once funding was in place we registered our project on PROSPERO, at that point we had run two rounds of searches (preliminary searches and one set to inform our funding application), and we had obtained IPD data from four studies. We

© 2022 Buckman JEJ et al. *JAMA Psychiatry.*

refined our searches by including other bibliographic databases and contacting other experts for missed studies, this helped us find further studies. We invited the chief investigators from each of those studies to join the project. We began to collect some further IPD from the studies that had agreed to take part. We then wrote up a protocol paper with information of what we would do with those IPD data once the dataset was complete. We ran further searches and found one more study just before initially submitting the protocol paper. The Protocol paper (including the searches) was peer-reviewed and we amended it post-review to give more details about this process. The protocol was then accepted for publication. It was amended once more when we decided that our choice of an I^2 threshold for considering problematic heterogeneity was too high, we dropped it from 80% to 75% in line with recommendations from Cochrane. We ran the final searches for studies meeting our inclusion criteria a few weeks before submitting this manuscript for publication and found no new studies meeting our criteria.

Our protocol paper provides information about all data we sought to extract from the included studies and all outcomes of interest. For the present study we were particularly interested in socioeconomic factors and potential confounders of the association between these factors and prognosis. We put together some exploratory directed acyclic graphs to consider what those confounders might be, and limited the data used for this study to those factors. Future studies using these data will consider the prognostic associations between other factors at baseline and prognosis. Further, for this study we amended our inclusion criteria slightly to exclude studies that did not include any measurement of socioeconomic factors at baseline, or that sampled only those in one category of any of those variables (e.g. all unemployed participants). There were two changes to the statistical analysis plan that should be noted: we did not include attrition as an outcome for the present study, and we also did not include conversions of scores on depressive symptom scales to the PROMIS T-score,[15] this was because here we included one study with a scale that could not be converted to the PROMIS (the GHQ-12).

eTable 2. Measures Used Across the RCTs Meeting Inclusion Criteria for the Present Study

**Measure**	**Details**	**Scores and Cut-offs for Remission**
The CIS-R[8]	Consists of 14 symptom subsections scored 0-4 covering core features of depression, depressive thoughts (scored 0-5), fatigue, concentration/forgetfulness, and sleep, generalized anxiety, worry, irritability, obsessions, compulsions, health anxiety, somatic concerns, phobic anxiety (split into agoraphobia, social phobia, and specific phobia), and panic. A final section measures general health, impairment and weight change.	The total score ranges from 0-57 with a cut-off of ≥12 used to indicate likely common mental disorder, primary and secondary diagnoses using ICD-10 criteria are given as are binary indictors of diagnosis for all the disorders assessed. The duration of each type of problem is also assessed for the present episode (or subsyndromal episode) up to the point of completing the CIS-R. Duration items are measured in five categories: 1) less than two weeks; 2) between two weeks and six months; 3) between six months and one year; 4) between one and two years; and 5) more than two years.
Beck Depression Inventory 2nd Edition (BDI-II)[16]	Consists of 21 items to assess depressive symptoms, each item is scored 0-3.	There is a maximum score obtainable of 63, and a cut-off of ≥10 is used indicate significant symptoms of depression, scores of <10 are therefore used to indicate remission in those that were previously depressed/scored ≥10.
Patient Health Questionnaire 9-item version (PHQ-9)[17]	This is a depression screening measure, with respondents asked to rate how often they have been bothered by each of the nine symptom items over the preceding two weeks. Each item is scored 0-3	There is a maximum score of 27 with a cut-off of ≥10 is used to indicate "caseness" for depression, a score of 9 or below for those that were previously depressed is therefore considered to indicate remission
General Health Questionnaire (12-item version) (GHQ-12)[18]	Consists of 12 items related to present and recent health over the "few weeks" prior to completion. Each item is related to depression or generalised anxiety, they are scored 0-0-1-1 for the four response options.	A cut-off of ≥2 is used to indicate the likely presence of common mental disorder, and so scores of <2 for those formally scoring above this would be considered to indicate remission
Social Support Scale - adapted by authors of RCTs	An 8-item instrument (the first seven of which are from the Health and Lifestyles Survey) assessing the degree to which participants rated the	N/A

© 2022 Buckman JEJ et al. *JAMA Psychiatry.*

[19] included in this IPD by adding one item to the Health and Lifestyles Survey Social Support Measure[20] .	social support of their friends and family in each of the following domains: 1) being accepted for who one is; 2) feeling cared about; 3) feeling loved; 4) feeling important to them; 5) being able to rely on them; 6) feeling well supported and encouraged by them; 7) being made to feel happy by them; and 8) feeling able to talk to them whenever one might like. Items are scored 1-3, with total scores ranging from 8-24; higher scores indicate higher levels of perceived social support. The authors of the Health and Lifestyles Survey suggested the maximum score for social support (which was 21 on that scale) indicated 'no lack of social support', scores between 18-20 indicated a 'moderate lack of social support', and scores of 17 or below indicated a 'severe lack of social support'.	
Life events: adapted by the authors of the Adult Psychiatric Morbidity Surveys [21] based on the Social Readjustment Rating Scale [22]	Participants are asked to respond yes/no to whether they have suffered any of eight events within the last six months e.g. a death/bereavement; being physically attacked/injured; or going through a divorce/separation. Each item is scored yes (1) or no (0) and the total score is the sum of all the items.	N/A
CIS-R was used in all studies n=4868. BDI-II was used in 6 studies (COBALT, GENPOD, IPCRESS, MIR, PANDA, & TREAD), n=2858 ; PHQ-9 was used in 5 studies (CADET, COBALT, MIR, PANDA, & REEACT) n=2807; GHQ was used in ITAS only n =796; EPDS was used in RESPOND only n =220; the Social Support Scale was used in 6 studies (COBALT, GENPOD, IPCRESS, MIR, PANDA, & TREAD) n =2858; and the Life Events Scale was used in 7 studies (COBALT, GENPOD, IPCRESS, ITAS, MIR, PANDA, & TREAD) n=3656		

© 2022 Buckman JEJ et al. *JAMA Psychiatry.*

Ethical Approvals and Trial Registrations details for the included RCTs

eTable 3. Ethical Approval and Trial Registration Details of the RCTs Meeting Inclusion Criteria for This Study

Study	Ethical Approvals	Trial Registration details
CADET	Granted by NHS Health Research Authority & NRES Committee South West (NRES/07/H1208/60)	ISRCTN32829227; https://doi.org/10.1186/ISRCTN32829227
COBALT	Approvals were granted by West Midlands Research Ethics Committee (NRES/07/H1208/60) and research governance approval was obtained from the local Primary Care Trusts/Health Boards	ISRCTN38231611; https://doi.org/10.1186/ISRCTN38231611
GENPOD	The South West Research Ethics Committee granted approval (MREC 02/6/076) and the Bristol, Manchester and Newcastle Primary Care NHS Trusts granted research governance approval.	ISRCTN31345163; https://doi.org/10.1186/ISRCTN31345163
IPCRESS	Approval granted by Royal Free and Hampstead Research Ethics Committee, reference number 05/Q0501/18	ISRCTN45444578; https://doi.org/10.1186/ISRCTN45444578
ITAS	Bro Taf Health Authority and United Bristol Healthcare Trust Local Research Ethics Committee	ISRCTN57116180; https://doi.org/10.1186/ISRCTN57116180
MIR	Approvals were granted by South East Wales Research Ethics Committee Panel C (ref: 12/WA/0353); Bristol Clinical Commissioning Group (CCG), and other CCGs provided research governance assurance.	ISRCTN06653773; https://doi.org/10.1186/ISRCTN06653773
PANDA	The Bristol Research Ethics Committee Centre granted ethics approval (12/SW/0267).	ISRCTN84544741; https://doi.org/10.1186/ISRCTN84544741
REEACT	The Leeds (East) research ethics committee granted approval (08/H1306/77).	ISRCTN91947481; https://doi.org/10.1186/ISRCTN91947481
TREAD	Approvals were granted by West Midlands multicentre research ethics committee (MREC 05/MRE07/42), and research governance approval was given by the relevant local National Health Service primary care trusts	ISRCTN16900744; https://doi.org/10.1186/ISRCTN16900744

© 2022 Buckman JEJ et al. *JAMA Psychiatry.*

Risk of Bias and Quality Ratings

eTable 4. QUIPS Risk of Bias Ratings

Study	Study Participation	Study Attrition	Prognostic Factor Measurement	Outcome Measurement	Study Confounding	Statistical Analysis and Reporting
CADET	Low	Moderate	Low	Low	Low	Low
COBALT	Low	Low	Low	Moderate	Low	Low
GENPOD	Low	Low	Low	Low	Low	Low
IPCRESS	Low	High	Low	Low	Low	Low
ITAS	Low	Low	Low	Low	Low	Low
MIR	Low	Moderate	Low	Low	Low	Low
PANDA	Low	Low	Low	Low	Low	Low
REEACT	Low	Moderate	Low	Low	Low	Low
TREAD	Low	Low	Low	Low	Low	Low

© 2022 Buckman JEJ et al. *JAMA Psychiatry.*

eTable 5. GRADE Quality Rating of Evidence for Each Type Prognostic Factor Assessed

		CADET	COBALT	GENPOD	IPCRESS	ITAS	MIR	PANDA	REEACT	TREAD	Overall Rating	Overall Quality Per Prognostic Factor
**Employment Status**	Risk of Bias	++++	++++	++++	++++	++++	++++	++++	++++	++++	++++	
	Imprecision	++++	++++	++++	++++	++++	++++	++++	++++	++++	++++	
	Inconsistency	++++	++++	++++	++++	++++	++++	++++	++++	++++	++++	
	Indirectness	N/A	N/A	N/A	N/A	N/A	N/A	N/A	N/A	N/A	++++	
	Publication Bias	N/A	N/A	N/A	N/A	N/A	N/A	N/A	N/A	N/A	++++	
	**OVERALL**											**HIGH**
**Financial Strain**	Risk of Bias	++++	++++	++++	++++	++++	++++	++++	++++	++++	++++	
	Imprecision	++++	++++	++++	++++	++++	++++	++++	++++	++++	++++	
	Inconsistency	++++	++++	++++	++++	++++	++++	++++	++++	++++	++++	
	Indirectness	N/A	N/A	N/A	N/A	N/A	N/A	N/A	N/A	N/A	++++	
	Publication Bias	N/A	N/A	N/A	N/A	N/A	N/A	N/A	N/A	N/A	++++	
	**OVERALL**											**HIGH**
**Housing Status**	Risk of Bias	++++	++++	++++	++++	++++	++++	++++	++++	++++	++++	
	Imprecision	++++	++++	++++	++++	++++	++++	++++	++++	++++	++++	
	Inconsistency	++++	++++	++++	++++	++++	++++	++++	++++	++++	++++	
	Indirectness	N/A	N/A	N/A	N/A	N/A	N/A	N/A	N/A	N/A	++++	
	Publication Bias	N/A	N/A	N/A	N/A	N/A	N/A	N/A	N/A	N/A	++++	
	**OVERALL**											**HIGH**
**Educational Attainment**	Risk of Bias	++++	++++	++++	++++	++++	++++	++++	++++	++++	++++	
	Imprecision	++++	++++	++++	++++	++++	++++	++++	++++	++++	++++	
	Inconsistency	++++	++++	++++	++++	++++	++++	++++	++++	++++	++++	
	Indirectness	N/A	N/A	N/A	N/A	N/A	N/A	N/A	N/A	N/A	++++	
	Publication Bias	N/A	N/A	N/A	N/A	N/A	N/A	N/A	N/A	N/A	++++	
	**OVERALL**											**HIGH**
**Overall Quality Per Study**		High	High	High	High	High	High	High	High	High		

These ratings were conducted independently by two reviewers, following recommendations [23–25] this table combines the outcomes from both. Ratings were made for each prognostic factor within each included study, across each study as a whole, and for each prognostic factor across all included studies. Ratings of indirectness and publication bias were only considered applicable for the prognostic factors across all included studies, not within any individual study.

© 2022 Buckman JEJ et al. *JAMA Psychiatry.*

eTable 6. Bibliographic Database Searches and Results for Preliminary Searches

Searches	Results
**Ovid MEDLINE 1860 to March 01 2016**	
1. exp major depression/ or exp "depression (emotion)"/	96427
2. exp Depressive Disorder, Major/	24516
3. exp Depressive Disorder, Major/ or exp Depressive Disorder/ or exp Depression/	183278
4. 1 or 2 or 3	183278
5. exp controlled clinical trial/ or exp "randomized controlled trial (topic)"/	529900
6. 4 and 5	14281
7. ("primary care" or "general practice" or "general practitioner pr GP").af.	163285
8. "Schedules for Clinical Assessment in Neuropsychiatry".af.	230
9. 6 and 7 and 8	**2**
10. ("structured clinical interview for DSM" or "SCID").af.	29843
11. 6 and 7 and 10	19
12. ("Clinical Interview Schedule" or "CIS-R" or "CISR" or "Revised clinical interview schedule" or "clinical interview schedule revised").af.	519
13. 6 and 7 and 12	12

© 2022 Buckman JEJ et al. *JAMA Psychiatry.*

eTable 7. Results From Database Searches and Results for Preliminary Searches and Reasons for Exclusion

Notes on potential for inclusion	Title	Journal and Citation	How Found	First Author
Protocol	Stop or go? Preventive cognitive therapy with guided tapering of antidepressants during pregnancy: study protocol of a pragmatic multicentre non-inferiority randomized controlled trial.	BMC Psychiatry. 16:72, 2016 Mar 18.	MEDLINE	Molenaar, Nina M
Not RCT	Cost-effectiveness of active monitoring versus antidepressants for major depression in primary health care: a 12-month non-randomized controlled trial (INFAP study).	BMC Psychiatry. 15:63, 2015 Mar 31.	MEDLINE	Rubio-Valera, Maria
Only Depression Module of SCID	ACTIVEDEP: a randomised, controlled trial of a home-based exercise intervention to alleviate depression in middle-aged and older adults.	British Journal of Sports Medicine. 48(3):226-32, 2014 Feb.	MEDLINE	Pfaff, Jon J
Only Depression Module of SCID	Training primary health care workers in mental health and its impact on diagnoses of common mental disorders in primary care of a developing country, Malawi: a cluster-randomized controlled trial.	Psychological Medicine. 44(3):657-66, 2014 Feb.	MEDLINE	Kauye, F
Protocol	Tavistock Adult Depression Study (TADS): a randomised controlled trial of psychoanalytic psychotherapy for treatment-resistant/treatment-refractory forms of depression.	BMC Psychiatry. 12:60, 2012 Jun 11.	MEDLINE	Taylor, David
Protocol	Cost-effectiveness of nurse-led self-help for recurrent depression in the primary care setting: design of a pragmatic randomised controlled trial.	BMC Psychiatry. 12:59, 2012 Jun 07.	MEDLINE	Biesheuvel-Leliefeld, Karolien E M
Not Depression	A randomized, controlled clinical trial: the effect of mindfulness-based cognitive therapy on generalized anxiety disorder among Chinese community patients: protocol for a randomized trial.	BMC Psychiatry. 11:187, 2011 Nov 29.	MEDLINE	Wong, Samuel Y S
Protocol	Brief cognitive behavioral therapy compared to general practitioners care for depression in primary care: a randomized trial.	Trials [Electronic Resource]. 11:96, 2010 Oct 12.	MEDLINE	Baas, Kim D
Protocol	Community pharmacist intervention in depressed primary care patients (PRODEFAR study): randomized controlled trial protocol.	BMC Public Health. 9:284, 2009 Aug 05.	MEDLINE	Rubio-Valera, Maria
Not RCT	SPIFA-A presentation of the Structured Psychiatric Interview for General Practice.	Nordic Journal of Psychiatry. 63(6):443-53, 2009 Nov.	MEDLINE	Dahl, Alv A
Older Adults Only and not full SCID	Reducing suicidal ideation in depressed older primary care patients.	Journal of the American Geriatrics Society. 54(10):1550-6, 2006 Oct.	MEDLINE	Unutzer, Jurgen
Not RCT	Performance of the PHQ-9 as a screening tool for depression after stroke.	Stroke. 36(3):635-8, 2005 Mar.	MEDLINE	Williams, Linda S
Older Adults Only and not full SCID	Low yield of thyroid-stimulating hormone testing in elderly patients with depression.	General Hospital Psychiatry. 26(4):302-9, 2004 Jul-Aug.	MEDLINE	Fraser, Shelagh A

© 2022 Buckman JEJ et al. *JAMA Psychiatry.*

Only Depression Module of SCID	Controlled trial of the short- and long-term effect of psychological treatment of post-partum depression. I. Impact on maternal mood.	British Journal of Psychiatry. 182:412-9, 2003 May.	MEDLINE	Cooper, Peter J
Not RCT	Long-term prognosis of depression in primary care.	Bulletin of the World Health Organization. 78(4):439-45, 2000.	MEDLINE	Simon, G E
Only Depression Module of SCID	Recovery from depression, work productivity, and health care costs among primary care patients.	General Hospital Psychiatry. 22(3):153-62, 2000 May-Jun.	MEDLINE	Simon, G E
Personality Disorder study and SCID for Axis-II only	Personality disorder comorbidity with major depression and response to treatment with sertraline or citalopram.	International Clinical Psychopharmacology. 13(5):205-11, 1998 Sep.	MEDLINE	Ekselius, L
Not RCT	Depressive disorders in primary care: prevalence, functional disability, and identification.	Journal of General Internal Medicine. 10(1):7-12, 1995 Jan.	MEDLINE	Williams, J W Jr
Not RCT	Physical symptom attributions: a defining characteristic of somatoform disorders?.	General Hospital Psychiatry. 37(2):147-52, 2015 Mar-Apr.	MEDLINE	Frostholm, Lisbeth
Not Depression RCT	Does psychological treatment help only those patients with severe irritable bowel syndrome who also have a concurrent psychiatric disorder?.	Australian & New Zealand Journal of Psychiatry. 39(9):807-15, 2005 Sep.	MEDLINE	Creed, Francis
Protocol	Mirtazapine added to selective serotonin reuptake inhibitors for treatment-resistant depression in primary care (MIR trial): study protocol for a randomised controlled trial.	Trials [Electronic Resource]. 17:66, 2016 Feb 03.	MEDLINE	Tallon, Debbie
Meets all inclusion criteria	Clinical effectiveness of collaborative care for depression in UK primary care (CADET): cluster randomised controlled trial.	BMJ. 347:f4913, 2013 Aug 19.	MEDLINE	Richards, David A
Meets all inclusion criteria	Facilitated physical activity as a treatment for depressed adults: randomised controlled trial.	BMJ. 344:e2758, 2012 Jun 06.	MEDLINE	Chalder, Melanie
Not all in primary care	Economic evaluation of a task-shifting intervention for common mental disorders in India.	Bulletin of the World Health Organization. 90(11):813-21, 2012 Nov 01.	MEDLINE	Buttorff, Christine
Protocol	Cognitive behavioural therapy as an adjunct to pharmacotherapy for treatment resistant depression in primary care: the CoBalT randomised controlled trial protocol.	Contemporary Clinical Trials. 33(2):312-9, 2012 Mar.	MEDLINE	Thomas, Laura J
Protocol	Physical activity as a treatment for depression: the TREAD randomised trial protocol.	Trials [Electronic Resource]. 11:105, 2010 Nov 12.	MEDLINE	Baxter, Helen
Meets all inclusion criteria	The prevalence of suicidal ideation identified by the Edinburgh Postnatal Depression Scale in postpartum women in primary care: findings from the RESPOND trial.	BMC Pregnancy & Childbirth. 11:57, 2011 Aug 03.	MEDLINE	Howard, Louise M
Meets all inclusion criteria	A trial of problem-solving by community mental health nurses for anxiety, depression and life difficulties among general practice patients. The CPN-GP study.	Health Technology Assessment (Winchester, England). 9(37):1-104, iii, 2005 Sep.	MEDLINE	Kendrick, T
Not RCT	Factors associated with being a false positive on the General Health Questionnaire.	Social Psychiatry & Psychiatric Epidemiology. 40(5):402-7, 2005 May.	MEDLINE	Bell, Truda

© 2022 Buckman JEJ et al. *JAMA Psychiatry.*

Meets all inclusion criteria	A randomised controlled trial to compare the cost-effectiveness of tricyclic antidepressants, selective serotonin reuptake inhibitors and lofepramine.	Health Technology Assessment (Winchester, England). 9(16):1-134, iii, 2005 May.	MEDLINE	Peveler, R
Meets all inclusion criteria	Computerised patient-specific guidelines for management of common mental disorders in primary care: a randomised controlled trial.	British Journal of General Practice. 54(508):832-7, 2004 Nov.	MEDLINE	Thomas, Hollie V
Meets all inclusion criteria	Randomised controlled trial of problem solving treatment, antidepressant medication, and combined treatment for major depression in primary care.	BMJ. 320(7226):26-30, 2000 Jan 01.	MEDLINE	Mynors-Wallis, L M
Not study of MDD - excluded those with MDD	Effect of a Web-Based Guided Self-help Intervention for Prevention of Major Depression in Adults With Subthreshold Depression: A Randomized Clinical Trial.	JAMA. 315(17):1854-63, 2016 May 03.	Contact with Experts	Buntrock, Claudia
Pilot study only	Anticipate: A pilot randomised trial of CBT for antenatal depression and validation of depression screening by midwives.	Archives of Women's Mental Health	Contact with Experts	Evans J.
Protocol	A written self-help intervention for depressed adults comparing behavioural activation combined with physical activity promotion with a self-help intervention based upon behavioural activation alone: study protocol for a parallel group pilot randomised controlled trial (BAcPAc).	Trials. 15 (pp 196), 2014. Date of Publication: 2014.	Contact with Experts	Farrand P.
Feasibility trial only	Antidepressant and group psychosocial treatment for depression: a rater blind exploratory RCT from a low income country.	Behavioural & Cognitive Psychotherapy. 42(6):693-705, 2014 Nov.	Contact with Experts	Husain N
Not Primary Care	A controlled study of fluoxetine and cognitive-behavioural counselling in the treatment of postnatal depression.	BMJ (Clinical research ed.), 1997, 314(7085), 932-936.	Contact with Experts	Appleby L
Older Adults Only and Protocol	A randomised evaluation of CollAborative care and active surveillance for Screen-Positive EldeRs with sub-threshold depression (CASPER): study protocol for a randomized controlled trial	Trials, 2011, 12, 225-234.	Contact with Experts	Mitchell, Natasha
Older Adults Only and Protocol	CASPER plus (CollAborative care in Screen-Positive EldeRs with major depressive disorder): study protocol for a randomised controlled trial	Trials, 2014, 15(1), 451-463	Contact with Experts	Overend, Karen
Not RCT	Outcomes of Depression International Network (ODIN)	British Journal of Psychiatry, 1998, 172(4), 359-363.	Contact with Experts	Dowrick, Christopher
Questionable for inclusion as only partially randomised - used full SCAN	Genome-wide association study of increasing suicidal ideation during antidepressant treatment in the GENDEP project	The Pharmacogenomics Journal, 2012, 12(1), 68-77.	Contact with Experts	Perroud, N
No comprehensive symptom or diagnostic screening measure; single self-report questionnaires only	Integrated primary care for patients with mental and physical multimorbidity: cluster randomised controlled trial of collaborative care for patients with depression comorbid with diabetes or cardiovascular disease	BMJ, 2015, 350:h638	Contact with Experts	Coventry, Peter

© 2022 Buckman JEJ et al. *JAMA Psychiatry.*

Pilot study only	A pilot randomised controlled trial of cognitive behavioural therapy for antenatal depression	BMC Psychiatry. 13:33, 2013.	Contact with Experts	Burns A
Not Primary Care	Cognitive Therapy vs Medications in the Treatment of Moderate to Severe Depression	Arch Gen Psych, 2005, 62(4), 409-416.	Hand Searching Reference Lists	Robert J. DeRubeis
Not Primary Care	Randomized Trial of Behavioral Activation , Cognitive Therapy , and Antidepressant Medication in the Acute Treatment of Adults With Major Depression	JCCP, 2006, 74(4)., 658-670.	Hand Searching Reference Lists	Dimidjian, Sona
Not Primary Care	Depression beliefs, treatment preference, and outcomes in a randomized trial for major depressive disorder	J Psych Res, 2012, 46(3), 375-381.	Hand Searching Reference Lists	Dunlop, Boadie W
Not Primary Care	COGNITIVE THERAPY VERSUS FLUOXETINE IN THE TREATMENT OF DYSTHYMIC DISORDER	Depression, 1996, 41(4), 34-41.	Hand Searching Reference Lists	Dunner, David L
Not Primary Care	National Institute of Mental Health Treatment of Depression Collaborative Research Program<br>General Effectiveness of Treatments	Arch Gen Psych, 1989, 46(11), 971-982.	Hand Searching Reference Lists	Irene Elkin
Used SCID data not presented but otherwise seems eligible	Effects of pharmacotherapy and psychotherapy in depressed primary-care patients: a randomized, controlled trial including a patients' choice arm	The International Journal of Neuropsychopharmacology, 2010, 13(1), 31	Hand Searching Reference Lists	Hegerl, Ulrich
Not Primary Care and no comprehensive measure of symptoms and disorders	Cognitive Therapy and Pharamacotherapy for Depression Singly and in Combination	Arch Gen Psych, 1992, 49, 774-781	Hand Searching Reference Lists	Hollon, Steven D.
Not Primary Care and no comprehensive measure of symptoms and disorders	Treatment of Atypical Depression With Cognitive Therapy or Phenelzine<br>A Double-blind, Placebo-Controlled Trial	Arch Gen Psychiatry. 1999;56(5):431-437.	Hand Searching Reference Lists	Robin B. Jarrett
Not Primary Care, unclear if full SCID or just Depression Module	Differences in Brain Glucose Metabolism Between Responders to CBT and Venlafaxine in a 16-Week Randomized Controlled Trial<br>Sidney	Am J Psychiatry 2007; 164:778–788	Hand Searching Reference Lists	Kennedy, Sidney H
Not Primary Care, no comprehensive measure of symptoms and disorders	Miranda J, Chung JY, Green BL et al. Treating depression in pre-dominantly low-income young minority women: a randomized controlled trial. JAMA 2003;290:57-65.<br>68.	JAMA, 2003, 290(1), 57-65	Hand Searching Reference Lists	Hegerl, Ulrich
Patients with Neurological Conditions	Comparative Outcomes for Individual Cognitive-Behavior Therapy, Supportive-Expressive Group Psychotherapy, and Sertraline for the Treatment of Depression in Multiple Sclerosis	JCCP, 2001, 69(6), 942-949	Hand Searching Reference Lists	Mohr, David C.
Not Primary Care	Cognitive Therapy and Pharmacotherapy<br>Singly and Together in the Treatment of Depression	Arch Gen Psychiatry. 1984;41(1):33-41	Hand Searching Reference Lists	George E. Murphy,
Not Primary Care and unclear if data recorded from psychiatric interviews	Comparative Efficacy of Cognitive Therapy and Pharmacotherapy in the Treatment of Depressed Outpatients	Cognitive Therapy and Research, 1977, 1(1), 17-37.	Hand Searching Reference Lists	Rush, Augustus J
Not Primary Care and unclear if full SCID	Cognitive reactivity to sad mood provocation and the prediction of depressive relapse.	Arch Gen Psychiatry, 2006, 63(7), 749-755.	Hand Searching Reference Lists	Segal, Zindel V

No limits or filters were set on searches, experts contacted included co-authors of this article and collaborators of theirs, hand searching of systematic reviews included [26,27].

© 2022 Buckman JEJ et al. *JAMA Psychiatry.*

Supplementary Results

eTable 8. Mean and SDs of the Overall *z* Score on the Depressive Symptom Measures at Baseline by Each Category of Each Socioeconomic Status Variable, Within Each Included Study

		Study																	
		CADET		COBALT		GENPOD		IPCRESS		ITAS		MIR		PANDA		REEACT		TREAD	
**Socioeconomic Variable**	**Factor**	**Mean**	**SD**	**Mean**	**SD**	**Mean**	**SD**	**Mean**	**SD**	**Mean**	**SD**	**Mean**	**SD**	**Mean**	**SD**	**Mean**	**SD**	**Mean**	**SD**
**Employment Status**	**Employed**	0.26	0.9	0	0.93	0.19	0.86	0.18	0.79	0	1	-0.06	0.87	-0.65	1.02	0.08	0.73	0.09	0.17
	**Not Seeking Employment**	0.24	0.95	-0.02	1.01	0.36	0.99	0.39	0.87	-0.13	0.98	-0.08	0.91	-0.83	1.01	0.17	0.76	0.17	0.86
	**Unemployed**	0.58	0.83	0.4	1.07	0.59	0.95	0.34	0.96	0.26	1.01	0.55	0.99	-0.36	1.04	0.46	0.78	0.41	0.82
**Financial Strain**	**Doing OK Financially**			-0.1	0.99	-0.01	0.86	-0.04	0.76	-0.14	0.98	-0.16	0.84	-0.92	0.94			0.12	0.94
	**Just about getting by**			0.19	0.97	0.24	0.88	0.45	0.78	-0.01	1.01	0.01	0.96	0.42	1.04			0.08	0.84
	**Struggling Financially**			0.36	1.05	0.67	0.88	0.53	0.86	0.27	0.98	0.46	0.95	-0.14	1.02			0.26	0.85
**Housing Status**	**Homeowner**	0.17	0.93			0.12	0.86	0.07	0.82	-0.05	1	-0.16	0.86	-0.95	0.97	0.09	0.76	-0.01	0.87
	**Tenant**	0.5	0.85			0.52	0.95	0.28	0.82	0.09	1.02	0.31	0.97	-0.5	1.06	0.31	0.74	0.26	0.9
	**Other Housing Status**	0.43	0.94			0.39	0.87	0.66	0.8	0.024	0.92	0.29	1.01	-0.4	0.93	0.1	0.75	0.42	0.72
**Educational Attainment**	**Degree or above**	-0.08	0.89	-0.13	0.88			0.16	0.81			-0.14	0.85	-0.72	1.05	0.05	0.74	0.01	0.8
	**A-level**	0.46	0.87	0.19	0.96			0.36	0.89			-0.01	0.91	-0.59	0.97	0.18	0.78	0.22	0.98
	**GCSE**	0.46	0.82	0.12	1.05			0.31	0.84			0.08	0.92	-0.7	0.99	0.23	0.73	0.17	0.86
	**No Formal Qualifications**	0.44	0.94	0.32	1.09			0.21	0.78			0.31	1.05	-0.6	1.22	0.82	1.05	0.22	0.83

Note: Where cells are blank studies did not collect data on the given variable

© 2022 Buckman JEJ et al. *JAMA Psychiatry.*

Secondary Outcomes

eTable 9. Difference in *z* Score of Depressive Symptoms at 6-8 Months After Baseline Per Unit Increase in Baseline Prognostic Indicator

	**Adjusted for treatment**			**Additionally adjusted for depressive severity and 'disorder characteristics'**			**Additionally adjusted for age, gender, and marital status**			**Additionally adjusted for employment status**		
**Baseline Variable**	**Z-score of depressive symptoms**	**Studies**	**Heterogeneity**	**Z-score of depressive symptoms**	**Studies**	**Heterogeneity**	**Z-score of depressive symptoms**	**Studies**	**Heterogeneity**	**Z-score of depressive symptoms**	**Studies**	**Heterogeneity**
	**Mean difference (95%CI)**	**K**	**I^2**	**Mean difference (95%CI)**	**K**	**I^2**	**Mean difference (95%CI)**	**K**	**I^2**	**Mean difference (95%CI)**	**K**	**I^2**
Employed (reference)	0			0			0			0		
Not Seeking Employment	0.07 (-0.04 to 0.17)	5	0	0.04 (-0.06 to 0.14)	5	0	0.06 (-0.05 to 0.17)	5	0			
Unemployed	0.59 (0.46 to 0.73)	5	8	0.39 (0.25 to 0.52)	5	18	0.36 (0.24 to 0.49)	5	0			
Financial Strain (Ordinal)	0.22 (0.11 to 0.33)	5	70	0.13 (0.05 to 0.21)	5	48	0.12 (0.04 to 0.19)	5	41	0.09 (0.02 to 0.16)	5	30
Doing OK Financially (reference)	0			0			0			0		
Just about getting by	0.16 (-0.04 to 0.36)	5	71	0.07 (-0.08 to 0.22)	5	53	0.06 (-0.09 to 0.20)	5	47	0.03 (-0.11 to 0.17)	5	44
Struggling Financially	0.44 (0.22 to 0.66)	5	70	0.26 (0.10 to 0.42)	5	48	0.24 (0.09 to 0.39)	5	40	0.18 (0.04 to 0.33)	5	31
Homeowner (reference)	0			0			0			0		
Tenant	0.31 (0.19 to 0.44)	4	18	0.20 (0.09 to 0.31)	4	2	0.21 (0.09 to 0.32)	4	0	0.17 (0.05 to 0.29)	4	0
Other Housing Status	0.38 (0.09 to 0.68)	4	64	0.21 (0.00 to 0.42)	4	34	0.20 (0.01 to 0.39)	4	0	0.16 (-0.03 to 0.35)	4	0
Educational Attainment (Ordinal)	0.10 (0.05 to 0.15)	4	0	0.05 (0.00 to 0.10)	4	0	0.06 (0.01 to 0.11)	4	0	0.04 (-0.03 to 0.10)	4	32
Degree or above (reference)	0			0			0			0		
A-level	0.08 (-0.07 to 0.22)	4	0	0.00 (-0.14 to 0.14)	4	0	-0.01 (-0.14 to 0.13)	4	0	-0.03 (-0.16 to 0.11)	4	0
GCSE	0.16 (0.00 to 0.31)	4	0	0.07 (-0.07 to 0.22)	4	4	0.08 (-0.07 to 0.24)	4	12	0.05 (-0.13 to 0.23)	4	35
No Formal Qualifications	0.31 (0.14 to 0.49)	4	0	0.16 (-0.01 to 0.32)	4	0	0.18 (0.02 to 0.35)	4	0	0.11 (-0.06 to 0.29)	4	1

Note: Association for ordinal variables is per category increase from first category shown below the variable down to the last (i.e. Doing OK financially, to just about getting by, to struggling financially). 'Disorder characteristics' adjusted for were baseline depressive symptom severity, average anxiety duration, depression duration, comorbid panic disorder, and history of antidepressant treatment.

eTable 10. Percentage Difference in Depressive Symptom Scale Scores at 6-8 Months After Baseline Per Unit Increase in Baseline Prognostic Indicator

	Adjusted for treatment			Additionally adjusted for depressive severity and 'disorder characteristics'			Additionally adjusted for age, gender, and marital status			Additionally adjusted for employment status		
**Baseline Variable**	**Percentage difference in depressive symptoms**	**Studies**	**Heterogeneity**	**Percentage difference in depressive symptoms**	**Studies**	**Heterogeneity**	**Percentage difference in depressive symptoms**	**Studies**	**Heterogeneity**	**Percentage difference in depressive symptoms**	**Studies**	**Heterogeneity**
	**% difference (95%CI)**	**K**	**I^2**	**% difference (95%CI)**	**K**	**I^2**	**% difference (95%CI)**	**K**	**I^2**	**% difference (95%CI)**	**K**	**I^2**
Employed (reference)	0			0			0			0		
Not Seeking Employment	8.2 (-1.1 to 18.3)	5	0	5.8 (-3.1 to 15.6)	5	0	7.5 (-2.4 to 18.4)	5	0			
Unemployed	52.2 (38.0 to 67.9)	5	0	32.2 (20.1 to 45.7)	5	0	30.2 (17.7 to 44.1)	5	0			
Financial Strain (Ordinal)	17.3 (8.4 to 27.0)	5	61	9.9 (2.6 to 17.7)	5	51	9.2 (1.6 to 17.4)	5	51	7.1 (-0.1 to 14.8)	5	45
Doing OK Financially (reference)	0			0			0			0		
Just about getting by	13.4 (-1.4 to 30.3)	5	55	7.1 (-3.6 to 18.9)	5	26	6.1 (-4.7 to 18.1)	5	26	4.4 (-5.9 to 15.7)	5	20
Struggling Financially	37.7 (17.5 to 61.4)	5	61	20.8 (5.1 to 38.9)	5	51	19.4 (3.4 to 38.0)	5	51	14.9 (-0.1 to 32.0)	5	45
Homeowner (reference)	0			0			0			0		
Tenant	27.0 (14.4 to 41.1)	4	0	15.5 (3.8 to 28.5)	4	8	14.2 (-2.0 to 33.2)	4	45	11.9 (-2.9 to 28.9)	4	32
Other Housing Status	38.5 (14.5 to 67.6)	4	30	21.4 (3.8 to 42.0)	4	0	19.5 (-0.4 to 43.4)	4	0	15.5 (-3.9 to 38.9)	4	0
Educational Attainment (Ordinal)	4.5 (0.3 to 8.9)	4	0	1.5 (-2.5 to 5.6)	1	0	2.4 (-2.1 to 7.0)	4	10	4.3 (-1.5 to 10.4)	4	0
Degree or above (reference)	0			0			0			0		
A-level	4.4 (-7.3 to 17.7)	4	0	-0.5 (-11.4 to 11.8)	4	0	-1.9 (-12.7 to 10.2)	4	0	-3.6 (-14.3 to 8.4)	4	0
GCSE	7.9 (-7.3 to 25.6)	4	30	2.0 (-11.8 to 17.9)	4	30	4.0 (-12.0 to 22.8)	4	46	1.2 (-16.0 to 22.0)	4	56
No Formal Qualifications	15.7 (1.4 to 32.1)	4	0	5.2 (-7.5 to 19.6)	4	0	7.1 (-6.3 to 22.4)	4	0	0.8 (-12.1 to 15.6)	4	0

Note: Association for ordinal variables is per category increase from first category shown below the variable down to the last (i.e. Doing OK financially, to just about getting by, to struggling financially). 'Disorder characteristics' adjusted for were baseline depressive symptom severity, average anxiety duration, depression duration, comorbid panic disorder, and history of antidepressant treatment.

© 2022 Buckman JEJ et al. *JAMA Psychiatry.*

eTable 11. Difference in *z* Score of Depressive Symptoms at 9-12 Months After Baseline Per Unit Increase in Baseline Prognostic Indicator

	Adjusted for treatment			Additionally adjusted for depressive severity and 'disorder characteristics'			Additionally adjusted for age, gender, and marital status			Additionally adjusted for employment status		
**Baseline Variable**	**Z-score of depressive symptoms**	**Studies**	**Heterogeneity**	**Z-score of depressive symptoms**	**Studies**	**Heterogeneity**	**Z-score of depressive symptoms**	**Studies**	**Heterogeneity**	**Z-score of depressive symptoms**	**Studies**	**Heterogeneity**
	**Mean difference (95%CI)**	**K**	**I^2**	**Mean difference (95%CI)**	**K**	**I^2**	**Mean difference (95%CI)**	**K**	**I^2**	**Mean difference (95%CI)**	**K**	**I^2**
Employed (reference)	0			0			0					
Not Seeking Employment	0.22 (0.09 to 0.34)	5	34	0.18 (0.07 to 0.29)	5	25	0.17 (0.07 to 0.27)	5	0			
Unemployed	0.62 (0.48 to 0.75)	5	31	0.39 (0.27 to 0.51)	5	18	0.37 (0.25 to 0.50)	5	19			
Financial Strain (Ordinal)	0.09 (-0.02 to 0.19)	3	50	0.01 (-0.06 to 0.08)	3	0	0.01 (-0.06 to 0.08)	3	0	-0.01 (-0.09 to 0.06)	5	0
Doing OK Financially (reference)	0			0			0			0		
Just about getting by	0.08 (-0.06 to 0.22)	3	0	0.02 (-0.11 to 0.15)	3	0	0.02 (-0.11 to 0.15)	3	0	0.00 (-0.13 to 0.13)	3	0
Struggling Financially	0.17 (-0.06 to 0.40)	3	57	0.02 (-0.12 to 0.16)	3	0	0.02 (-0.13 to 0.17)	3	0	-0.04 (-0.19 to 0.11)	3	1
Homeowner (reference)	0			0			0			0		
Tenant	0.33 (0.17 to 0.49)	4	57	0.19 (0.08 to 0.29)	4	17	0.22 (0.11 to 0.33)	4	0	0.14 (0.03 to 0.25)	4	0
Other Housing Status	0.25 (-0.05 to 0.55)	4	69	0.14 (-0.10 to 0.37)	4	58	0.21 (-0.06 to 0.49)	4	59	0.14 (-0.14 to 0.42)	4	60
Educational Attainment (Ordinal)	0.13 (0.09 to 0.17)	5	0	0.08 (0.04 to 0.12)	5	0	0.08 (0.04 to 0.12)	5	0	0.05 (0.01 to 0.09)	5	0
Degree or above (reference)	0			0			0			0		
A-level	0.14 (0.02 to 0.26)	5	0	0.02 (-0.09 to 0.13)	5	0	0.03 (-0.09 to 0.14)	5	0	0.01 (-0.11 to 0.12)	5	0
GCSE	0.17 (0.06 to 0.28)	5	0	0.07 (-0.04 to 0.17)	5	0	0.07 (-0.03 to 0.18)	5	0	0.03 (-0.07 to 0.14)	5	0
No Formal Qualifications	0.49 (0.34 to 0.63)	5	0	0.30 (0.16 to 0.43)	5	0	0.28 (0.14 to 0.42)	5	0	0.20 (0.05 to 0.34)	5	0

Note: Association for ordinal variables is per category increase from first category shown below the variable down to the last (i.e. Doing OK financially, to just about getting by, to struggling financially). 'Disorder characteristics' adjusted for were baseline depressive symptom severity, average anxiety duration, depression duration, comorbid panic disorder, and history of antidepressant treatment.

© 2022 Buckman JEJ et al. *JAMA Psychiatry.*

eTable 12. Percentage Difference in Depressive Symptom Scale Scores at 9-12 Months After Baseline Per Unit Increase in Baseline Prognostic Indicator

	**Adjusted for treatment**			**Additionally adjusted for depressive severity and 'disorder characteristics'**			**Additionally adjusted for age, gender, and marital status**			**Additionally adjusted for employment status**		
**Baseline Variable**	**Percentage difference in depressive symptoms**	**Studies**	**Heterogeneity**	**Percentage difference in depressive symptoms**	**Studies**	**Heterogeneity**	**Percentage difference in depressive symptoms**	**Studies**	**Heterogeneity**	**Percentage difference in depressive symptoms**	**Studies**	**Heterogeneity**
	**% difference (95%CI)**	**K**	**I^2**	**% difference (95%CI)**	**K**	**I^2**	**% difference (95%CI)**	**K**	**I^2**	**% difference (95%CI)**	**K**	**I^2**
Employed (reference)	0			0			0			0		
Not Seeking Employment	26.3 (13.9 to 40.1)	4	4	22.9 (10.9 to 36.3)	4	9	21.7 (9.4 to 35.2)	4	3			
Unemployed	60.3 (44.3 to 78.1)	4	0	36.5 (22.7 to 51.8)	4	0	33.8 (19.7 to 49.5)	4	0			
Financial Strain (Ordinal)	8.1 (1.3 to 15.3)	3	0	1.8 (-4.3 to 8.2)	3	0	1.9 (-4.5 to 8.7)	3	0	-0.4 (-6.7 to 6.4)	3	0
Doing OK Financially (reference)	0			0			0			0		
Just about getting by	7.5 (-5.2 to 21.9)	3	0	2.9 (-8.7 to 15.9)	3	0	2.9 (-8.8 to 16.0)	3	0	1.9 (-9.6 to 14.9)	3	0
Struggling Financially	16.8 (2.5 to 33.0)	3	0	3.4 (-8.6 to 17.1)	3	0	3.9 (-8.7 to 18.2)	3	0	-0.9 (-13.1 to 13.1)	3	0
Homeowner (reference)	0			0			0			0		
Tenant	24.0 (10.5 to 39.1)	3	2	12.4 (0.6 to 25.6)	3	0	15.6 (2.9 to 30.0)	3	0	8.6 (-3.9 to 22.7)	3	0
Other Housing Status	16.2 (-16.5 to 61.7)	3	70	5.3 (-21.8 to 41.7)	3	65	9.4 (-25.1 to 59.8)	3	73	3.0 (-28.8 to 48.9)	3	71
Educational Attainment (Ordinal)	7.1 (2.7 to 11.7)	4	0	4.0 (-0.1 to 8.4)	4	0	3.5 (-0.7 to 8.0)	4	0	-0.4 (-6.7 to 6.4)	4	0
Degree or above (reference)	0			0			0			0		
A-level	12.0 (-0.7 to 26.3)	4	0	6.1 (-5.5 to 19.2)	4	0	6.0 (-5.6 to 19.1)	4	0	4.6 (-6.9 to 17.5)	4	0
GCSE	6.7 (-4.6 to 19.2)	4	0	1.7 (-8.7 to 13.3)	4	0	2.2 (-8.3 to 13.9)	4	0	-0.7 (-10.9 to 10.7)	4	0
No Formal Qualifications	36.5 (17.1 to 59.1)	4	0	22.0 (5.3 to 41.5)	4	0	18.5 (1.8 to 37.8)	4	0	9.8 (-5.8 to 27.9)	4	0

Note: Association for ordinal variables is per category increase from first category shown below the variable down to the last (i.e. Doing OK financially, to just about getting by, to struggling financially). 'Disorder characteristics' adjusted for were baseline depressive symptom severity, average anxiety duration, depression duration, comorbid panic disorder, and history of antidepressant treatment.

© 2022 Buckman JEJ et al. *JAMA Psychiatry.*

eTable 13. Odds Ratios for Being in Remission at 3-4 Months After Baseline Per Unit Increase in Baseline Prognostic Indicator

	Adjusted for treatment			Additionally adjusted for depressive severity and 'disorder characteristics'			Additionally adjusted for age, gender, and marital status			Additionally adjusted for employment status		
**Baseline Variable**	**Odds ratio for being in Remission at 3-4 months**	**Studies**	**Heterogeneity**	**Odds ratio for being in Remission at 3-4 months**	**Studies**	**Heterogeneity**	**Odds ratio for being in Remission at 3-4 months**	**Studies**	**Heterogeneity**	**Odds ratio for being in Remission at 3-4 months**	**Studies**	**Heterogeneity**
	**Odds Ratio (95%CI)**	**K**	**I^2**	**Odds Ratio (95%CI)**	**K**	**I^2**	**Odds Ratio (95%CI)**	**K**	**I^2**	**Odds Ratio (95%CI)**	**K**	**I^2**
Employed (reference)	0			0			0			0		
Not Seeking Employment	0.96 (0.92 to 1.01)	8	32	0.97 (0.93 to 1.01)	8	15	0.97 (0.93 to 1.01)	8	0			
Unemployed	0.83 (0.78 to 0.88)	8	0	0.89 (0.85 to 0.95)	8	46	0.90 (0.85 to 0.95)	8	0			
Financial Strain (Ordinal)	0.72 (0.65 to 0.81)	6	0	0.87 (0.77 to 0.97)	6	0	0.88 (0.78 to 0.99)	6	0	0.91 (0.81 to 1.04)	6	0
Doing OK Financially (reference)	0			0			0			0		
Just about getting by	0.93 (0.89 to 0.97)	6	0	0.98 (0.94 to 1.02)	6	0	0.99 (0.94 to 1.03)	6	0	0.99 (0.95 to 1.03)	6	0
Struggling Financially	0.87 (0.83 to 0.91)	6	0	0.94 (0.90 to 0.99)	6	0	0.95 (0.90 to 1.00)	6	0	0.96 (0.92 to 1.01)	6	0
Homeowner (reference)	0			0			0			0		
Tenant	0.90 (0.87 to 0.93)	7	0	0.95 (0.92 to 0.98)	7	0	0.95 (0.91 to 0.99)	7	0	0.96 (0.93 to 1.00)	7	0
Other Housing Status	0.86 (0.80 to 0.92)	7	0	0.90 (0.85 to 0.95)	7	29	0.90 (0.85 to 0.96)	7	0	0.92 (0.86 to 0.97)	7	0
Educational Attainment (Ordinal)	0.84 (0.72 to 0.98)	7	75	0.88 (0.75 to 1.03)	7	73	0.87 (0.75 to 1.01)	7	67	0.89 (0.78 to 1.02)	7	55
Degree or above (reference)	0			0			0			0		
A-level	0.93 (0.88 to 0.99)	7	37	0.96 (0.92 to 1.01)	7	0	0.97 (0.93 to 1.01)	7	0	0.97 (0.93 to 1.02)	7	0
GCSE	0.91 (0.83 to 1.00)	7	74	0.94 (0.87 to 1.02)	7	66	0.94 (0.87 to 1.02)	7	64	0.95 (0.89 to 1.02)	7	56
No Formal Qualifications	0.88 (0.78 to 0.99)	7	75	0.93 (0.84 to 1.03)	7	67	0.92 (0.84 to 1.01)	7	57	0.94 (0.87 to 1.02)	7	34

Note: Association for ordinal variables is per category increase from first category shown below the variable down to the last (i.e. Doing OK financially, to just about getting by, to struggling financially). 'Disorder characteristics' adjusted for were baseline depressive symptom severity, average anxiety duration, depression duration, comorbid panic disorder, and history of antidepressant treatment.

© 2022 Buckman JEJ et al. *JAMA Psychiatry.*

Sensitivity Analyses

eTable 14. Difference in *z* Score of Depressive Symptoms at 3-4 Months After Baseline Per Unit Increase in Baseline Prognostic Indicator, Adjusting for Variables That Were Systematically Missing in Some Studies

	Adjusted for treatment, 'disorder characteristics', age, gender, marital status, employment status, and housing status^			^ and additionally adjusted for Long-term health condition status			^ and additionally adjusted for highest level of educational attainment			Adjusted for treatment, 'disorder characteristics', age, gender, marital status, employment status, and financial strain*			* and additionally adjusted social support		
**Baseline Variable**	**Z-score of depressive symptoms**	**Studies**	**Heterogeneity**	**Z-score of depressive symptoms**	**Studies**	**Heterogeneity**	**Z-score of depressive symptoms**	**Studies**	**Heterogeneity**	**Z-score of depressive symptoms**	**Studies**	**Heterogeneity**	**Z-score of depressive symptoms**	**Studies**	**Heterogeneity**
	**Mean difference (95%CI)**	**K**	**I^2**	**Mean difference (95%CI)**	**K**	**I^2**	**Mean difference (95%CI)**	**K**	**I^2**	**Mean difference (95%CI)**	**K**	**I^2**	**Mean difference (95%CI)**	**K**	**I^2**
Employed (reference)	0			0			0			0			0		
Not Seeking Employment	0.03 (-0.05 to 0.11)	7	0	0.02 (-0.06 to 0.10)	7	0	0.02 (-0.07 to 0.10)	6	0	0.03 (-0.05 to 0.12)	6	49	0.03 (-0.06 to 0.12)	6	0
Unemployed	0.26 (0.17 to 0.36)	7	0	0.26 (0.16 to 0.35)	7	0	0.23 (0.12 to 0.34)	6	0	0.27 (0.11 to 0.43)	6	49	0.27 (0.10 to 0.43)	6	51
Financial Strain (Ordinal)	0.06 (0.01 to 0.12)	5	0	0.06 (0.01 to 0.12)	5	0	0.05 (-0.01 to 0.12)	4	0				0.05 (0.00 to 0.10)	6	0
Doing OK Financially (reference)	0			0			0						0		
Just about getting by	0.03 (-0.06 to 0.13)	5	0	0.03 (-0.06 to 0.13)	5	0	0.03 (-0.08 to 0.13)	4	0				0.04 (-0.04 to 0.13)	6	0
Struggling Financially	0.14 (0.02 to 0.25)	5	0	0.14 (0.02 to 0.25)	5	0	0.11 (-0.02 to 0.25)	4	0				0.10 (0.00 to 0.20)	6	0
Homeowner (reference)				0			0			0			0		
Tenant				0.11 (0.03 to 0.19)	7	0	0.10 (0.01 to 0.18)	6	0	0.09 (-0.01 to 0.19)	5	0	0.09 (-0.01 to 0.19)	5	0
Other Housing Status				0.20 (0.08 to 0.32)	7	0	0.19 (0.05 to 0.33)	6	0	0.19 (0.04 to 0.34)	5	0	0.17 (0.02 to 0.33)	5	0
Educational Attainment (Ordinal)	0.05 (0.01 to 0.09)	6	18	0.05 (0.01 to 0.09)	6	17				0.02 (-0.03 to 0.07)	4	0	0.02 (-0.03 to 0.07)	4	0
Degree or above (reference)	0			0						0			0		
A-level	-0.04 (-0.13 to 0.05)	6	0	-0.04 (-0.13 to 0.05)	6	0				-0.03 (-0.14 to 0.08)	4	0	-0.03 (-0.14 to 0.08)	4	0
GCSE	0.05 (-0.04 to 0.15)	6	0	0.05 (-0.04 to 0.15)	6	0				-0.01 (-0.14 to 0.11)	4	0	-0.01 (-0.14 to 0.11)	4	0
No Formal Qualifications	0.14 (0.00 to 0.27)	6	0	0.13 (0.00 to 0.27)	6	0				0.09 (-0.08 to 0.25)	4	0	0.08 (-0.08 to 0.25)	4	0

Note: Association for ordinal variables is per category increase from first category shown below the variable down to the last (i.e. Doing OK financially, to just about getting by, to struggling financially). 'Disorder characteristics' adjusted for are: baseline depressive symptom severity, average anxiety duration, depression duration, comorbid panic disorder, and history of antidepressant treatment.

eTable 15. Percentage Difference in Depressive Symptom Scale Scores at 3-4 Months After Baseline Per Unit Increase in Baseline Prognostic Indicator Adjusting for Variables That Were Systematically Missing in Some Studies

© 2022 Buckman JEJ et al. *JAMA Psychiatry.*

	Adjusted for treatment, 'disorder characteristics', age, gender, marital status, employment status, and housing status^			^ and additionally adjusted for Long-term health condition status			^ and additionally adjusted for highest level of educational attainment			Adjusted for treatment, 'disorder characteristics', age, gender, marital status, employment status, and financial strain*			* and additionally adjusted social support		
**Baseline Variable**	**Percentage difference in depressive symptoms**	**Studies**	**Heterogeneity**	**Percentage difference in depressive symptoms**	**Studies**	**Heterogeneity**	**Percentage difference in depressive symptoms**	**Studies**	**Heterogeneity**	**Percentage difference in depressive symptoms**	**Studies**	**Heterogeneity**	**Percentage difference in depressive symptoms**	**Studies**	**Heterogeneity**
	**% difference (95%CI)**	**K**	**I^2**	**% difference (95%CI)**	**K**	**I^2**	**% difference (95%CI)**	**K**	**I^2**	**% difference (95%CI)**	**K**	**I^2**	**% difference (95%CI)**	**K**	**I^2**
Employed (reference)	0			0			0			0			0		
Not Seeking Employment	6.1 (-1.1 to 13.8)	7	0	5.3 (-1.8 to 13.0)	7	0	5.8 (-2.0 to 14.2)	6	0	6.5 (-0.9 to 14.4)	6	0	6.3 (-1.0 to 14.2)	6	0
Unemployed	21.5 (12.5 to 31.2)	7	0	20.8 (11.8 to 30.4)	7	0	18.9 (9.3 to 29.4)	6	0	24.4 (14.8 to 34.8)	6	1	23.4 (13.5 to 34.2)	6	6
Financial Strain (Ordinal)	3.3 (-1.7 to 8.5)	5	0	3.2 (-1.8 to 8.4)	5	0	0.9 (-4.6 to 6.7)	4	0				1.8 (-2.2 to 6.0)	6	0
Doing OK Financially (reference)	0			0			0						0		
Just about getting by	2.3 (-6.1 to 11.3)	5	0	2.4 (-5.9 to 11.5)	5	0	1.2 (-7.9 to 11.1)	4	0				2.5 (-4.3 to 9.8)	6	0
Struggling Financially	7.2 (-3.1 to 18.6)	5	0	6.9 (-3.4 to 18.3)	5	0	2.0 (-9.1 to 14.4)	4	0				3.7 (-4.4 to 12.5)	6	0
Homeowner (reference)	0			0			0			0			0		
Tenant				9.2 (1.9 to 17.0)	7	0	8.9 (1.0 to 17.4)	6	0	6.6 (-2.4 to 16.5)	5	0	6.5 (-2.5 to 16.4)	5	0
Other Housing Status				17.2 (6.0 to 29.6)	7	0	18.1 (5.9 to 31.7)	6	0	16.2 (2.7 to 31.5)	5	0	15.2 (1.8 to 30.4)	5	0
Educational Attainment (Ordinal)	1.7 (-2.3 to 5.9)	6	36	1.7 (-2.3 to 5.8)	6	35				-1.3 (-5.4 to 2.9)	4	0	-1.4 (-5.4 to 2.9)	4	0
Degree or above (reference)	0			0			0			0			0		
A-level	-2.9 (-10.4 to 5.3)	6	0	-2.7 (-10.3 to 5.6)	6	0				-2.6 (-11.7 to 7.5)	4	0	-2.3 (-11.6 to 7.8)	4	0
GCSE	0.8 (-9.3 to 12.0)	6	38	0.6 (-9.3 to 11.7)	6	36				-6.1 (-16.6 to 5.8)	4	11	-6.3 (-16.2 to 4.8)	4	2
No Formal Qualifications	3.9 (-7.2 to 16.4)	6	0	3.8 (-7.4 to 16.3)	6	0				-0.1 (-13.2 to 15.0)	4	0	-0.2 (-13.3 to 14.9)	4	0

Note: Association for ordinal variables is per category increase from first category shown below the variable down to the last (i.e. Doing OK financially, to just about getting by, to struggling financially). 'Disorder characteristics' adjusted for were baseline depressive symptom severity, average anxiety duration, depression duration, comorbid panic disorder, and history of antidepressant treatment.

eTable 16. Difference in z Score of Depressive Symptom Scale Scores at 6-8 Months After Baseline Per Unit Increase in Baseline Prognostic Indicator Adjusting for Variables That Were Systematically Missing in Some Studies

© 2022 Buckman JEJ et al. *JAMA Psychiatry.*

	Adjusted for treatment, 'disorder characteristics', age, gender, marital status, employment status, and housing status^			^ and additionally adjusted for Long-term health condition status			^ and additionally adjusted for highest level of educational attainment			Adjusted for treatment, 'disorder characteristics', age, gender, marital status, employment status, and financial strain*			* and additionally adjusted social support		
**Baseline Variable**	**Z-score of depressive symptoms**	**Studies**	**Heterogeneity**	**Z-score of depressive symptoms**	**Studies**	**Heterogeneity**	**Z-score of depressive symptoms**	**Studies**	**Heterogeneity**	**Z-score of depressive symptoms**	**Studies**	**Heterogeneity**	**Z-score of depressive symptoms**	**Studies**	**Heterogeneity**
	**Mean difference (95%CI)**	**K**	**I^2**	**Mean difference (95%CI)**	**K**	**I^2**	**1Mean difference (95%CI)**	**K**	**I^2**	**Mean difference (95%CI)**	**K**	**I^2**	**Mean difference (95%CI)**	**K**	**I^2**
Employed (reference)	0			0			0			0			0		
Not Seeking Employment	0.00 (-0.12 to 0.12)	4	0	0.00 (-0.12 to 0.12)	4	0	0.00 (-0.16 to 0.16)	3	0	0.05 (-0.06 to 0.16)	5	0	0.07 (-0.07 to 0.20)	4	0
Unemployed	0.24 (0.09 to 0.40)	4	0	0.25 (0.10 to 0.4)	4	0	0.21 (0.02 to 0.40)	3	0	0.32 (0.19 to 0.45)	5	0	0.33 (0.17 to 0.49)	4	7
Financial Strain (Ordinal)	0.08 (0.00 to 0.17)	4	42	0.08 (-0.01 to 0.17)	4	48	0.05 (-0.06 to 0.16)	3	44				0.06 (-0.01 to 0.13)	4	9
Doing OK Financially (reference)	0			0			0						0		
Just about getting by	0.01 (-0.15 to 0.17)	4	46	0.01 (-0.17 to 0.18)	4	52	-0.05 (-0.26 to 0.17)	3	50				-0.01 (-0.18 to 0.16)	4	44
Struggling Financially	0.16 (-0.01 to 0.34)	4	43	0.16 (-0.03 to 0.35)	4	49	0.10 (-0.13 to 0.33)	3	46				0.12 (-0.03 to 0.27)	4	11
Homeowner (reference)				0			0			0			0		
Tenant				0.17 (0.05 to 0.28)	4	0	0.12 (-0.03 to 0.27)	3	0	0.13 (0.01 to 0.25)	4	0	0.09 (-0.07 to 0.26)	3	9
Other Housing Status				0.15 (-0.04 to 0.35)	4	0	0.18 (-0.09 to 0.44)	3	19	0.15 (-0.05 to 0.34)	4	0	0.17 (-0.08 to 0.43)	3	11
Educational Attainment (Ordinal)	0.06 (-0.01 to 0.12)	3	0	0.05 (-0.01 to 0.12)	3	0				0.05 (-0.01 to 0.12)	3	0	0.05 (-0.01 to 0.12)	3	0
Degree or above (reference)	0			0						0			0		
A-level	0.00 (-0.16 to 0.16)	3	0	-0.01 (-0.18 to 0.15)	3	0				0.00 (-0.16 to 0.16)	3	11	0.00 (-0.16 to 0.16)	3	0
GCSE	0.09 (-0.11 to 0.30)	3	31	0.08 (-0.11 to 0.28)	3	23				0.08 (-0.10 to 0.26)	3	11	0.08 (-0.10 to 0.25)	3	8
No Formal Qualifications	0.17 (-0.03 to 0.38)	3	0	0.17 (-0.04 to 0.37)	3	0				0.17 (-0.04 to 0.37)	3	0	0.17 (-0.04 to 0.37)	3	0

Note: Association for ordinal variables is per category increase from first category shown below the variable down to the last (i.e. Doing OK financially, to just about getting by, to struggling financially). 'Disorder characteristics' adjusted for were baseline depressive symptom severity, average anxiety duration, depression duration, comorbid panic disorder, and history of antidepressant treatment.

eTable 17. Percentage Difference in Depressive Symptom Scale Scores at 6-8 Months After Baseline Per Unit Increase in Baseline Prognostic Indicator Adjusting for Variables That Were Systematically Missing in Some Studies

© 2022 Buckman JEJ et al. *JAMA Psychiatry.*

	Adjusted for treatment, 'disorder characteristics', age, gender, marital status, employment status, and housing status^			^ and additionally adjusted for Long-term health condition status			^ and additionally adjusted for highest level of educational attainment			Adjusted for treatment, 'disorder characteristics', age, gender, marital status, employment status, and financial strain*			* and additionally adjusted social support		
**Baseline Variable**	**Percentage difference in depressive symptoms**	**Studies**	**Heterogeneity**	**Percentage difference in depressive symptoms**	**Studies**	**Heterogeneity**	**Percentage difference in depressive symptoms**	**Studies**	**Heterogeneity**	**Percentage difference in depressive symptoms**	**Studies**	**Heterogeneity**	**Percentage difference in depressive symptoms**	**Studies**	**Heterogeneity**
	**% difference (95%CI)**	**K**	**I^2**	**% difference (95%CI)**	**K**	**I^2**	**% difference (95%CI)**	**K**	**I^2**	**% difference (95%CI)**	**K**	**I^2**	**% difference (95%CI)**	**K**	**I^2**
Employed (reference)	0			0			0			0			0		
Not Seeking Employment	1.4 (-10.0 to 14.1)	4	0	0.9 (-10.4 to 13.6)	4	0	2.7 (-12.2 to 20.2)	3	0	7.0 (-2.8 to 17.8)	5	0	8.3 (-4.5 to 22.9)	4	14
Unemployed	22.0 (6.4 to 39.8)	4	0	22.7 (7.1 to 40.6)	4	0	16.7 (-1.4 to 38.2)	3	0	25.9 (13.5 to 39.8)	5	0	24.1 (10.3 to 39.5)	4	0
Financial Strain (Ordinal)	6.7 (-3.0 to 17.3)	4	0	6.6 (-3.1 to 17.3)	4	56	2.3 (-6.7 to 12.1)	3	27				3.7 (-2.2 to 6.0)	4	0
Doing OK Financially (reference)	0			0			0						0		
Just about getting by	4.2 (-9.1 to 19.3)	4	29	4.2 (-9.5 to 19.9)	4	33	0.1 (-16.8 to 20.4)	3	37				0.9 (-10.0 to 13.1)	4	12
Struggling Financially	13.9 (-5.9 to 37.9)	4	55	13.8 (-6.2 to 38.0)	4	57	4.4 (-12.9 to 25.1)	3	25				7.6 (-4.1 to 20.7)	4	0
Homeowner (reference)	0			0			0			0			0		
Tenant				11.8 (-2.8 to 28.6)	4	30	5.8 (-12.1 to 27.4)	3	37	8.9 (-5.9 to 26.1)	4	33	4.1 (-15.7 to 28.7)	3	49
Other Housing Status				15.2 (-4.1 to 38.5)	4	0	13.6 (-9.3 to 42.2)	3	0	14.0 (-5.3 to 37.2)	4	0	12.8 (-9.8 to 40.9)	3	0
Educational Attainment (Ordinal)	3.5 (0.2 to 6.9)	3	0	3.0 (-2.8 to 9.3)	3	0				3.0 (-2.9 to 9.2)	3	0	2.9 (-3.0 to 9.2)	3	0
Degree or above (reference)	0			0						0			0		
A-level	-2.2 (-16.5 to 14.5)	3	0	-2.9 (-17.2 to 13.9)	3	0				-2.1 (-16.5 to 14.8)	3	0	-2.4 (-16.4 to 14.9)	3	0
GCSE	7.6 (-13.2 to 33.4)	3	43	6.8 (-12.6 to 30.4)	3	35				6.2 (-12.7 to 29.1)	3	31	5.7 (-12.5 to 27.8)	3	26
No Formal Qualifications	8.9 (-9.8 to 31.5)	3	0	8.8 (-9.9 to 31.4)	3	0				8.8 (-10.0 to 31.4)	3	0	8.7 (-10.0 to 31.3)	3	0

Note: Association for ordinal variables is per category increase from first category shown below the variable down to the last (i.e. Doing OK financially, to just about getting by, to struggling financially). 'Disorder characteristics' adjusted for were baseline depressive symptom severity, average anxiety duration, depression duration, comorbid panic disorder, and history of antidepressant treatment.

eTable 18. Difference in *z* Score of Depressive Symptoms at 9-12 Months After Baseline Per Unit Increase in Baseline Prognostic Indicator Adjusting for Variables That Were Systematically Missing in Some Studies

© 2022 Buckman JEJ et al. *JAMA Psychiatry.*

	Adjusted for treatment, 'disorder characteristics', age, gender, marital status, employment status, and housing status^			^ and additionally adjusted for Long-term health condition status			^ and additionally adjusted for highest level of educational attainment			Adjusted for treatment, 'disorder characteristics', age, gender, marital status, employment status, and financial strain*			* and additionally adjusted social support		
**Baseline Variable**	**Z-score of depressive symptoms**	**Studies**	**Heterogeneity**	**Z-score of depressive symptoms**	**Studies**	**Heterogeneity**	**Z-score of depressive symptoms**	**Studies**	**Heterogeneity**	**Z-score of depressive symptoms**	**Studies**	**Heterogeneity**	**Z-score of depressive symptoms**	**Studies**	**Heterogeneity**
	**Mean difference (95%CI)**	**K**	I^2	**Mean difference (95%CI)**	**K**	I^2	**Mean difference (95%CI)**	**K**	I^2	**Mean difference (95%CI)**	**K**	I^2	**Mean difference (95%CI)**	**K**	I^2
Employed (reference)	0			0			0			0			0		
Not Seeking Employment	0.12 (0.00 to 0.24)	4	17	0.11 (-0.01 to 0.24)	4	15	0.10 (-0.03 to 0.22)	4	20	0.20 (0.04 to 0.36)	3	20	0.20 (0.04 to 0.35)	3	19
Unemployed	0.31 (0.14 to 0.47)	1	33	0.30 (0.14 to 0.46)	4	28	0.28 (0.10 to 0.46)	4	40	0.31 (0.09 to 0.54)	3	48	0.32 (0.09 to 0.56)	3	53
Financial Strain (Ordinal)	0.01 (-0.09 to 0.10)	2	0	0.01 (-0.08 to 0.11)	2	0	0.01 (-0.08 to 0.11)	2	0				-0.02 (-0.10 to 0.06)	3	13
Doing OK Financially (reference)	0			0			0						0		
Just about getting by	-0.03 (-0.20 to 0.14)	2	0	-0.03 (-0.19 to 0.14)	2	0	-0.04 (-0.20 to 0.13)	2	0				0.01 (-0.12 to 0.14)	3	0
Struggling Financially	0.02 (-0.17 to 0.21)	2	0	0.02 (-0.17 to 0.22)	2	0	0.03 (-0.17 to 0.22)	2	0				-0.06 (-0.24 to 0.13)	3	35
Homeowner (reference)	0			0			0			0			0		
Tenant				0.14 (0.03 to 0.25)	4	0	0.12 (0.01 to 0.23)	4	0	0.13 (-0.03 to 0.30)	2	0	0.13 (-0.04 to 0.3)	2	0
Other Housing Status				0.14 (-0.14 to 0.41)	4	59	0.12 (-0.16 to 0.39)	4	57	0.17 (-0.53 to 0.87)	2	83	0.17 (-0.54 to 0.88)	2	83
Educational Attainment (Ordinal)	0.06 (0.01 to 0.10)	4	0	0.06 (0.01 to 0.10)	4	0				0.06 (-0.01 to 0.13)	2	0	0.06 (-0.01 to 0.13)	2	0
Degree or above (reference)	0			0						0			0		
A-level	-0.02 (-0.15 to 0.10)	4	0	-0.03 (-0.15 to 0.10)	4	0				0.00 (-0.19 to 0.19)	2	0	0.00 (-0.19 to 0.19)	2	0
GCSE	0.03 (-0.09 to 0.14)	4	0	0.03 (-0.09 to 0.14)	4	0				0.00 (-0.18 to 0.19)	2	0	0.00 (-0.19 to 0.19)	2	0
No Formal Qualifications	0.20 (0.03 to 0.37)	4	0	0.20 (0.03 to 0.37)	4	0				0.22 (0.00 to 0.45)	2	0	0.22 (0.00 to 0.45)	2	0

Note: Association for ordinal variables is per category increase from first category shown below the variable down to the last (i.e. Doing OK financially, to just about getting by, to struggling financially). 'Disorder characteristics' adjusted for were baseline depressive symptom severity, average anxiety duration, depression duration, comorbid panic disorder, and history of antidepressant treatment.

eTable 19. Percentage Difference in Depressive Symptom Scale Scores at 9-12 Months After Baseline Per Unit Increase in Baseline Prognostic Indicator Adjusting for Variables That Were Systematically Missing in Some Studies

© 2022 Buckman JEJ et al. *JAMA Psychiatry.*

	**Adjusted for treatment, 'disorder characteristics', age, gender, marital status, employment status, and housing status^**			**^ and additionally adjusted for Long-term health condition status**			**^ and additionally adjusted for highest level of educational attainment**			**Adjusted for treatment, 'disorder characteristics', age, gender, marital status, employment status, and financial strain***			*** and additionally adjusted social support**		
**Baseline Variable**	**Percentage difference in depressive symptoms**	**Studies**	**Heterogeneity**	**Percentage difference in depressive symptoms**	**Studies**	**Heterogeneity**	**Percentage difference in depressive symptoms**	**Studies**	**Heterogeneity**	**Percentage difference in depressive symptoms**	**Studies**	**Heterogeneity**	**Percentage difference in depressive symptoms**	**Studies**	**Heterogeneity**
	**% difference (95%CI)**	**K**	**I^2**	**% difference (95%CI)**	**K**	**I^2**	**% difference (95%CI)**	**K**	**I^2**	**% difference (95%CI)**	**K**	**I^2**	**% difference (95%CI)**	**K**	**I^2**
Employed (reference)	0			0			0			0			0		
Not Seeking Employment	15.9 (-0.8 to 35.5)	3	29	15.1 (-1.5 to 34.4)	3	28	14.4 (-3.0 to 35.0)	3	36	22.2 (3.7 to 44.1)	3	33	22.1 (3.7 to 43.8)	3	31
Unemployed	27.5 (9.7 to 48.3)	3	0	26.5 (8.8 to 47.1)	3	0	26.2 (8.5 to 46.9)	3	0	33.5 (16.8 to 52.6)	3	0	34.0 (17.3 to 53.0)	3	0
Financial Strain (Ordinal)	-1.5 (-10.5 to 8.4)	2	0	-1.5 (-10.5 to 8.4)	2	0	-1.4 (-10.4 to 8.5)	2	0				-1.0 (-7.3 to 5.7)	3	0
Doing OK Financially (reference)	0			0			0						0		
Just about getting by	-3.2 (-19.0 to 15.5)	2	0	-3.3 (-19.1 to 15.5)	2	0	-3.8 (-19.5 to 15.0)	2	0				2.0 (-9.6 to 15.0)	3	0
Struggling Financially	-2.7 (-19.7 to 18.0)	2	0	-2.8 (-19.8 to 17.9)	2	0	-2.5 (-19.5 to 18.2)	2	0				-2.3 (-14.3 to 11.5)	3	0
Homeowner (reference)	0			0			0			0			0		
Tenant				8.4 (-4.1 to 22.5)	3	0	7.7 (-4.8 to 21.8)	3	0	12.6 (-5.6 to 34.4)	2	0	12.6 (-5.7 to 34.5)	2	0
Other Housing Status				3.2 (-28.4 to 48.5)	3	70	3.1 (-29.0 to 49.7)	3	71	4.5 (-48.7 to 112.8)	2	85	4.7 (-48.8 to 114.0)	2	85
Educational Attainment (Ordinal)	2.6 (-0.8 to 6.1)	3	0	1.8 (-3.5 to 7.4)	3	0				1.1 (-6.0 to 8.8)	2	0	1.1 (-6.0 to 8.8)	2	0
Degree or above (reference)	0			0						0			0		
A-level	1.9 (-11.8 to 17.6)	3	0	1.4 (-12.3 to 17.1)	3	0				-3.0 (-20.6 to 18.5)	2	0	-3.0 (-20.6 to 18.5)	2	0
GCSE	0.3 (-11.6 to 13.8)	3	0	0.4 (-11.5 to 14.0)	3	0				-5.4 (-22.6 to 15.5)	2	0	-5.7 (-22.8 to 15.3)	2	0
No Formal Qualifications	9.6 (-11.5 to 35.9)	3	0	9.6 (-11.6 to 35.7)	3	0				6.9 (-14.9 to 34.3)	2	0	7.0 (-14.8 to 34.4)	2	0

Note: Association for ordinal variables is per category increase from first category shown below the variable down to the last (i.e. Doing OK financially, to just about getting by, to struggling financially). 'Disorder characteristics' adjusted for were baseline depressive symptom severity, average anxiety duration, depression duration, comorbid panic disorder, and history of antidepressant treatment.

© 2022 Buckman JEJ et al. *JAMA Psychiatry.*

eTable 20. Odds Ratios for Being in Remission at 3-4 Months After Baseline Per Unit Increase in Baseline Prognostic Indicator, Adjusting for Variables That Were Systematically Missing in Some Studies

	**Adjusted for treatment, 'disorder characteristics', age, gender, marital status, employment status, and housing status^**			**^ and additionally adjusted for Long-term health condition status**			**^ and additionally adjusted for highest level of educational attainment**			**Adjusted for treatment, 'disorder characteristics', age, gender, marital status, employment status, and financial strain***			*** and additionally adjusted social support**		
**Baseline Variable**	**Odds ratio for being in Remission at 3-4 months**	**Studies**	**Heterogeneity**	**Odds ratio for being in Remission at 3-4 months**	**Studies**	**Heterogeneity**	**Odds ratio for being in Remission at 3-4 months**	**Studies**	**Heterogeneity**	**Odds ratio for being in Remission at 3-4 months**	**Studies**	**Heterogeneity**	**Odds ratio for being in Remission at 3-4 months**	**Studies**	**Heterogeneity**
	**Odds Ratio (95%CI)**	**K**	**I^2**	**Odds Ratio (95%CI)**	**K**	**I^2**	**Odds Ratio (95%CI)**	**K**	**I^2**	**Odds Ratio (95%CI)**	**K**	**I^2**	**Odds Ratio (95%CI)**	**K**	**I^2**
Employed (reference)	0			0			0			0			0		
Not Seeking Employment	0.99 (0.95 to 1.03)	7	0	0.99 (0.95 to 1.04)	7	0	1.00 (0.95 to 1.05)	6	0	0.97 (0.93 to 1.02)	6	0	0.98 (0.93 to 1.02)	6	1
Unemployed	0.92 (0.88 to 0.97)	7	7	0.93 (0.88 to 0.97)	7	3	0.95 (0.90 to 1.00)	6	0	0.92 (0.86 to 0.99)	6	49	0.93 (0.86 to 1.00)	6	50
Financial Strain (Ordinal)	0.89 (0.77 to 1.03)	5	0	0.89 (0.78 to 1.03)	5	0	0.91 (0.77 to 1.08)	4	0				0.93 (0.82 to 1.06)	6	0
Doing OK Financially (reference)	0			0			0						0		
Just about getting by	0.99 (0.94 to 1.04)	5	0	0.99 (0.94 to 1.04)	5	0	0.99 (0.93 to 1.04)	4	0				0.99 (0.95 to 1.04)	6	0
Struggling Financially	0.95 (0.90 to 1.01)	5	0	0.95 (0.90 to 1.01)	5	0	0.96 (0.90 to 1.02)	4	0				0.97 (0.92 to 1.02)	6	0
Homeowner (reference)				0			0			0			0		
Tenant				0.97 (0.93 to 1.01)	7	0	0.96 (0.92 to 1.01)	6	0	0.98 (0.93 to 1.03)	5	0	0.98 (0.93 to 1.03)	5	0
Other Housing Status				0.92 (0.86 to 0.97)	7	0	0.91 (0.85 to 0.97)	6	0	0.92 (0.86 to 0.99)	5	5	0.93 (0.86 to 0.99)	5	0
Educational Attainment (Ordinal)	0.92 (0.78 to 1.08)	6	61	0.92 (0.78 to 1.08)	6	61				1.02 (0.86 to 1.22)	4	42	1.03 (0.86 to 1.22)	4	41
Degree or above (reference)	0			0						0			0		
A-level	0.99 (0.94 to 1.04)	6	0	0.99 (0.94 to 1.04)	6	0				1.00 (0.95 to 1.06)	4	0	1.00 (0.94 to 1.06)	4	0
GCSE	0.97 (0.89 to 1.05)	6	63	0.97 (0.89 to 1.05)	6	63				1.02 (0.93 to 1.11)	4	45	1.02 (0.93 to 1.11)	4	44
No Formal Qualifications	0.97 (0.88 to 1.06)	6	36	0.97 (0.89 to 1.06)	6	35				1.01 (0.92 to 1.10)	4	20	1.01 (0.92 to 1.10)	4	18

Note: Association for ordinal variables is per category increase from first category shown below the variable down to the last (i.e. Doing OK financially, to just about getting by, to struggling financially). 'Disorder characteristics' adjusted for were baseline depressive symptom severity, average anxiety duration, depression duration, comorbid panic disorder, and history of antidepressant treatment.

eTable 21. Results of Original Analyses and Corresponding Sensitivity Analyses Removing Studies Due to Heterogeneity

© 2022 Buckman JEJ et al. *JAMA Psychiatry.*

Prognostic Variable	Analysis	Pooled Effect Estimate
Educational Attainment (Ordinal)		**Odds Ratio (95%CI)**
	Original analysis of association with remission at 3-4 months post-baseline adjusted for treatment only	0.84 (0.72 to 0.98)
	Analysis removing study contributing most to heterogeneity (MIR)	0.79 (0.70 to 0.89)
No Formal Qualifications	Original analysis of association with remission at 3-4 months post-baseline adjusted for treatment only	0.88 (0.78 to 0.99)
	Analysis removing study contributing most to heterogeneity (MIR)	0.84 (0.76 to 0.94)
Other Housing Status		**Mean difference (95%CI)**
	Original analysis of association with z-score at 9-12 months post-baseline adjusted for treatment, 'disorder characteristics', age, gender, marital status, employment status, and financial strain	0.17 (-0.53 to 0.87)
	Analysis by study as only two studies contributed to the meta-analysis	0.53 (0.13 to 0.93) and -0.19 (-0.62 to 0.23)
	Original analysis of association with z-score at 9-12 months post-baseline adjusted for treatment, 'disorder characteristics', age, gender, marital status, employment status, and financial strain and social support	0.17 (-0.54 to 0.88)
	Analysis by study as only two studies contributed to the meta-analysis	0.53 (0.13 to 0.93) and -0.20 (-.62 to 0.23)
		**% difference (95%CI)**
	Original analysis of association with log outcome at 9-12 months post-baseline adjusted for treatment, 'disorder characteristics', age, gender, marital status, employment status, and financial strain	4.5 (-48.7 to 112.8)
	Analysis by study as only two studies contributed to the meta-analysis	48.4 (4.7 to 110.2) and -28.2 (-53.6 to 11.2)
	Original analysis of association with log outcome at 9-12 months post-baseline adjusted for treatment, 'disorder characteristics', age, gender, marital status, employment status, and financial strain and social support	4.7 (-48.8 to 114.0)
	Analysis by study as only two studies contributed to the meta-analysis	48.9 (5.2 to 110.6) and -28.3 (-53.8 to 11.3)

© 2022 Buckman JEJ et al. *JAMA Psychiatry.*

© 2022 Buckman JEJ et al. *JAMA Psychiatry*.

eReferences

1. Button KS, Kounali D, Thomas L, et al. Minimal clinically important difference on the Beck Depression Inventory - II according to the patient's perspective. *Psychol Med*. 2015;45(15):3269-3279. doi:10.1017/S0033291715001270

2. Horton NJ, Lipsitz SR. Multiple Imputation in Practice: Comparison of Software Packages for Regression Models With Missing Variables. *Am Stat*. 2001;55(3):244-254. doi:10.1198/000313001317098266

3. Royston P, White IR. Multiple Imputation by Chained Equations (MICE): Implementation in Stata. *J Stat Softw*. 2011;45(4):1-20. doi:10.1002/mpr.329.Multiple

4. Stata Press. Stata Statistical Software: Release 16. StataCorp LLC.

5. Fisher DJ. Two-stage individual participant data meta-analysis and generalized forest plots. *Stata J*. 2015;2:369-396.

6. Royston P. Multiple imputation of missing values: Further update of ice, with an emphasis on categorical variables. *Stata J*. 2009;9(3):466-477.

7. Morris TP, White IR, Royston P. Tuning multiple imputation by predictive mean matching and local residual draws. *BMC Med Res Methodol*. 2014;14(1):75. doi:10.1186/1471-2288-14-75

8. Lewis G, Pelosi AJ, Araya R, Dunn G. Measuring psychiatric disorder in the community : a standardized assessment for use by lay interviewers. *Psychol Med*. 1992;22:465-486.

9. Wing JK, Barbor T, Brugha TS, et al. SCAN. *Arch Gen Psychiatry*. 1990;47(6):589-593. doi:10.1001/archpsyc.1990.01810180089012

10. Perroud N, Uher R, Ng MYM, et al. Genome-wide association study of increasing suicidal ideation during antidepressant treatment in the GENDEP project. *Pharmacogenomics J*. 2012;12(1):68-77. doi:10.1038/tpj.2010.70

11. Williams J, Gibbon M, First M, et al. The structured clinical interview for DSM-III-R (SCID): II. Multisite test-retest reliability. *Arch Gen Psychiatry*. 1992;49(8):630-636.

12. Hegerl U, Hautzinger M, Mergl R, et al. Effects of pharmacotherapy and psychotherapy in depressed primary-care patients: a randomized, controlled trial including a patients' choice arm. *Int J Neuropsychopharmacol*. 2010;13(01):31-44. doi:10.1017/S1461145709000224

13. Lesko CR, Jacobson LP, Althoff KN, et al. Collaborative, pooled and harmonized study designs for epidemiologic research: Challenges and opportunities. *Int J Epidemiol*. 2018;47(2):654-668. doi:10.1093/IJE/DYX283

14. Buckman JEJ, Saunders R, Cohen ZD, et al. The contribution of depressive 'disorder characteristics' to determinations of prognosis for adults with depression: an individual patient data meta-analysis. *Psychol Med*. 2021;51(7):1068-1081. doi:10.1017/S0033291721001367

15. Choi SW, Schalet BD, Cook KF, Cella D. Establishing a common metric for depressive symptoms: Linking the BDI-II , CES-D, and PHQ-9 to PROMIS Depression. *Psychol Assess*. 2014;26(2):513-527. doi:10.1037/a0035768

16. Beck AT, Steer RA, Brown GK. *Manual for the Beck Depression Inventory-II*. San Antonio, Tx: Psychological Corporation; 1996.

17. Kroenke K, Spitzer RL, Williams JBW. The PHQ-9. *J Gen Intern Med*. 2001;16(9):606-613. doi:10.1046/j.1525-1497.2001.016009606.x

18. Goldberg D. General Health Questionnaire (GHQ-12) NFER. 1992.

19. Kessler D, Lewis G, Kaur S, et al. Therapist-delivered internet psychotherapy for depression in primary care: a randomised controlled trial. *Lancet*. 2009;374(9690):628-634. doi:10.1016/S0140-6736(09)61257-5

20. Cox BD, Blaxter M, Buckle ALJ, et al. *The Health and Lifestyle Survey*. London: Health Promotion

© 2022 Buckman JEJ et al. *JAMA Psychiatry.*

Research Trust; 1987.

21. Goh KEJ, Simmons MY, Hamilton AR. Electron-electron interactions in highly disordered two-dimensional systems. McManus S, Bebbington P, Jenkins R, Brugha T, eds. *Phys Rev B*. 2008;77(23):235410. doi:10.1103/PhysRevB.77.235410

22. Holmes TH, Rahe RH. The social readjustment rating scale. *J Psychosom Res*. 1967;11(2):213-218.

23. Guyatt GH, Oxman AD, Vist GE, et al. GRADE: an emerging consensus on rating quality of evidence and strength of recommendations. *BMJ*. 2008;336(7650):924-926. doi:10.1136/bmj.39489.470347.AD

24. Siemieniuk R, Guyatt GH. What is GRADE? *BMJ best Pract*. 2019.

25. Balshem H, Helfand M, Schünemann HJ, et al. GRADE guidelines: 3. Rating the quality of evidence. *J Clin Epidemiol*. 2011;64(4):401-406. doi:10.1016/j.jclinepi.2010.07.015

26. Weitz ES, Hollon SD, Twisk J, et al. Baseline depression severity as moderator of depression outcomes between cognitive behavioral therapy vs pharmacotherapy: An individual patient data meta-analysis. *JAMA Psychiatry*. 2015;72(11):1102-1109. doi:10.1001/jamapsychiatry.2015.1516

27. Cuijpers P, Weitz E, Twisk J, et al. Gender as predictor and moderator of outcome in cognitive behavior therapy and pharamcotherapy for adult depression: An "individual patient data" meta-analysis. *Depress Anxiety*. 2014;31:941-951. doi:10.1002/da.22328

© 2022 Buckman JEJ et al. *JAMA Psychiatry.*
